# Near-Infrared Light-Driven Microgrooved UCNPs/Azobenzene-LCE Actuators and Substrates for Cardiomyoblast Alignment

**DOI:** 10.1021/acsami.6c07815

**Published:** 2026-07-04

**Authors:** Chun Li, Zhenjia Huang, Tongqing Li, Petar S. Uskoković, Mo Yang, Linxia Gu, Gary Chi-Pong Tsui

**Affiliations:** † Advanced Manufacturing Technology Research Centre, Department of Industrial and Systems Engineering, 26680 The Hong Kong Polytechnic University, Hong Kong, China; ‡ Faculty of Technology and Metallurgy, University of Belgrade, Karnegijeva 4, Belgrade 11120, Serbia; § Department of Biomedical Engineering, The Hong Kong Polytechnic University, Hong Kong, China; ∥ Department of Biomedical Engineering and Science, Florida Institute of Technology, Melbourne, Florida 32901, United States

**Keywords:** liquid crystal elastomer, upconversion nanoparticles, near-infrared actuation, azobenzene photoisomerization, microgroove topography, cell alignment

## Abstract

Photoisomerization-based liquid crystal elastomer (LCE) actuators offer precise spatiotemporal control over mechanical deformation, yet their reliance on ultraviolet (UV) irradiation limits biomedical applicability due to phototoxicity and poor tissue penetration. Herein, we report an 808 nm near-infrared (NIR) light-driven photoisomerization actuator based on azobenzene-cross-linked LCEs (Azo-LCEs) integrated with NaYF_4_:Yb/Tm@NaYF_4_:Yb/Nd@NaYF_4_ core–shell–shell upconversion nanoparticles (CSS-UCNPs). The Nd^3+^-sensitized CSS-UCNPs convert 808 nm NIR light into UV/blue upconversion emissions (345–476 nm), driving *trans*-to-*cis* isomerization of azobenzene units and inducing macroscopic bending of the LCE films. To evaluate the actuation performance, the UCNPs/Azo-LCE films were tested under continuous-wave 808 nm irradiation for 20 s at power densities of 4–24 W cm^–2^, reaching a maximum bending angle of 42.8 ± 2.6° at 24 W cm^–2^ and exhibiting stable cyclic actuation over 50 cycles at 16 W cm^–2^. For biologically relevant operation, thermal assessment at lower irradiation intensities revealed only a limited temperature rise in the culture medium under 4–8 W cm^–2^ irradiation (Δ*T* = 2.29–4.36 °C), with no obvious cumulative heating during cyclic operation (20 s on/20 s off, 50 cycles). In addition, microgroove-patterned UCNPs/Azo-LCE substrates supported the adhesion and spreading of rat cardiomyoblast cells (H9c2) and guided groove-width-dependent uniaxial alignment. This work establishes a strategy for 808 nm-excited photoisomerization-driven LCE actuation and highlights its potential as an NIR-addressable soft actuator platform for future studies of dynamic cell-guidance, while operating with a modest thermal burden under the tested conditions.

## Graphical abstract

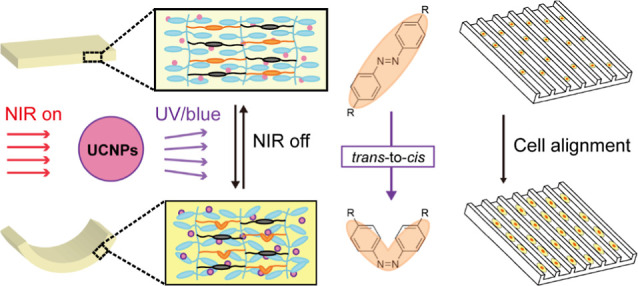

## 1 Introduction

In native myocardium, cardiomyocytes are continuously subjected to cyclic mechanical loading arising from the heart’s rhythmic contraction. A substantial body of evidence has demonstrated that recapitulating such dynamic mechanical cues in vitro is important for promoting the structural and functional maturation of engineered cardiac tissues. Cyclic mechanical conditioning has been shown to enhance sarcomere organization, promote cardiomyocyte hypertrophy, and improve contractile synchrony in three-dimensional cardiac constructs. To achieve this, conventional approaches often employ bioreactor systems to apply cyclic strain to cells cultured on elastic membranes.^1^ However, these systems are typically bulky, operationally complex, and lack the capacity for spatially precise stimulation. These limitations have motivated the exploration of remotely actuatable smart substrates that can deliver programmable mechanical cues without direct physical contact,^2^ among which light-driven actuators are particularly attractive owing to their inherent advantages of noninvasive operation and high spatiotemporal controllability.

Among the various photoresponsive polymer materials, including hydrogels,^3,4^ shape memory polymers (SMPs),^5,6^ and liquid crystal elastomers (LCEs), LCEs have emerged as particularly promising candidates. Their unique combination of the entropy elasticity of polymer networks with the orientational anisotropy of liquid crystalline mesogens enables large-amplitude, reversible shape morphing under light illumination, with the additional advantages of remote noncontact operation and precise spatiotemporal control.^7–9^ Light-driven LCEs can be classified into two categories based on the rearrangement of mesogens induced by different triggering factors under light stimulation: photoisomerization-based actuation and photothermal-based actuation. Photothermal LCEs incorporate nanoscale heat transducers, such as carbon-based nanomaterials,^10,11^ gold nanorods,^12–15^ or conjugated polymers^16,17^ —that convert absorbed light into localized heat, raising the temperature above the nematic-to-isotropic transition temperature (*T*_NI_) to trigger mesogen disorder and macroscopic deformation. Although this mechanism enables NIR-responsive actuation with appreciable tissue penetration, the accompanying local temperature elevation can cause irreversible damage to cells, severely limiting its applicability in biomedical contexts.^15,18^ In contrast, photoisomerization-based LCEs exploit the conformational switching of photochromic moieties—most commonly azobenzene derivatives—to disrupt mesogen order directly via a photochemical pathway, without requiring a significant temperature rise.^15^ This athermal actuation mechanism is potentially more attractive for biological environments.

Azobenzene-containing LCEs are fabricated by chemically incorporating azobenzene units into the LCE network as main-chain segments,^19,20^ cross-linkers^21–23^ or pendant groups.^24–27^ Upon UV irradiation (350–400 nm), the azobenzene moieties undergo *trans*-to-*cis* isomerization, disrupting mesogen order and producing macroscopic strains including bending, contraction, and twisting. The reverse *cis*-to-*trans* transition occurs under visible light or thermal relaxation, restoring the original shape and enabling cyclic actuation. However, the reliance on UV light poses fundamental limitations: UV radiation suffers from shallow penetration depth in most polymeric^24^ and biological matrices, can cause photodamage to living cells and tissues, and restricts practical deployment in scenarios requiring deep or safe light delivery.

Near-infrared (NIR) light within the first biological transparency window (700–1100 nm) offers significantly deeper tissue penetration and lower photodamage, making it an ideal excitation source for biomedical actuators.^28–30^ Lanthanide-doped upconversion nanoparticles (UCNPs),^31–33^ which convert NIR photons into higher-energy UV/visible emissions through sequential energy transfer processes, provide an elegant bridge between NIR excitation and azobenzene photoisomerization. Coupled with their reported biocompatibility and low cytotoxicity in appropriately engineered formulations, UCNPs offer a promising avenue for constructing biosafe photoresponsive materials.^34–36^ By selecting UCNPs whose emission spectra overlap with the π–π* absorption band of azobenzene, NIR-driven photoisomerized LCE actuation becomes feasible. Wu et al. demonstrated this concept using Yb^3+^/Tm^3+^-doped UCNPs coated on azobenzene-containing LCE films, realizing reversible photomechanical bending under 980 nm NIR irradiation.^37^ However, since this pioneering work, further development of UCNP-mediated photomechanical LCE actuators has been limited. UCNP-azobenzene combinations have instead been extensively pursued for optical applications, including photoswitching,^35,36,38^ photonic tuning,^39–41^ and fluorescence modulation,^42^ while their potential for driving programmable macroscopic deformation in LCE networks—and particularly for applications that demand both deep light penetration and biological safety—remains underexplored.

In addition to dynamic mechanical stimulation, substrate surface topography constitutes another critical biophysical cue that governs cardiomyocyte organization. In the native myocardium, the extracellular matrix presents highly anisotropic fibrillar architectures that direct cell elongation and alignment along preferred fiber orientations. In vitro studies have demonstrated that microfabricated surface patterns—such as grooves, ridges, and aligned fibers—can recapitulate this contact guidance effect, promoting cardiomyocyte alignment, elongation, and organized sarcomeric assembly.^43–47^ An ideal substrate for cardiac tissue engineering would therefore combine programmable mechanical actuation with topographical guidance within a single integrated platform, providing both dynamic and static biophysical cues to direct cardiomyocyte organization.

Herein, we report an 808 nm NIR-responsive photoisomerization actuator system based on azobenzene-containing LCEs (Azo-LCEs) integrated with NaYF_4_:20%Yb/0.5%Tm@NaYF_4_:10%Yb/20%Nd@NaYF_4_ core–shell–shell UCNPs (CSS-UCNPs). The CSS-UCNPs feature a Tm^3+^-doped core emitting at 345–476 nm that spectrally overlaps with the azobenzene π–π* absorption band, a Yb^3+^/Nd^3+^ codoped intermediate shell enabling efficient 808 nm excitation through a Nd^3+^→Yb^3+^→Tm^3+^ energy cascade, and an inert NaYF_4_ outer shell to suppress surface quenching and enhance upconversion luminescence efficiency.^48–50^ This strategic integration enables rapid and repeatable photomechanical bending under NIR irradiation while maintaining modest temperature increases during operation. Furthermore, microgroove-patterned substrates based on this composite can guide cellular alignment. This work therefore presents a promising material system for near-infrared-triggered soft actuation and for static micropattern-guided cell alignment, with potential for future studies on dynamic cell guidance.

## 2 Experimental Section

### 2.1 Preparation of Liquid Crystal Cells for Liquid Crystal Array Arrangement

Liquid crystal (LC) cells were fabricated to ensure uniaxial alignment of LC mesogens. Two glass substrates were sequentially ultrasonicated in deionized water and acetone, followed by nitrogen blow-drying. The cleaned substrates were spin-coated with the PI-based alignment agent DL-2590 at 1000 rpm for 1 min using a desktop spin coater. After spin-coating, the substrates were prebaked on a hot plate at 100 °C for 5 min and subsequently cured at 200 °C for 30 min. Uniaxial planar alignment was induced by repeated unidirectional rubbing of the coated surfaces with a velvet cloth. An LC cell was assembled by sandwiching two rubbed glass substrates with their coated surfaces facing each other and rubbing directions aligned in a parallel configuration, separated by 30 μm spacers.

### 2.2 Preparation Procedure of LCE Films

The preparation of photoresponsive Azo-LCEs is illustrated in Figure 1. The LC mixtures were prepared by first ultrasonically dissolving LC monomer C6BP, cross-linker RM257, photoinitiator Irgacure 369, and the Azo in acetone, followed by dispersing UCNPs in the same solvent, with all components in varying ratios. The resulting mixtures were then slowly stirred on a heating platform at 65 °C to completely evaporate the acetone solvent and form the LC mixtures. The LC mixtures were injected onto the edge of the LC cells and slowly infiltrated into the cell along the rubbing direction on a hot plate at 65 °C. The cell was cooled down to room temperature at a rate of 5 °C·min^–1^ and then exposed to a UV lamp (6 W, λ = 365 nm) for 1 h to complete photopolymerization. Finally, after opening the cell, the Azo-LCE film was peeled off and cut into strips of 10 mm × 2 mm × 30 μm along the rubbing direction.

**Figure 1:**
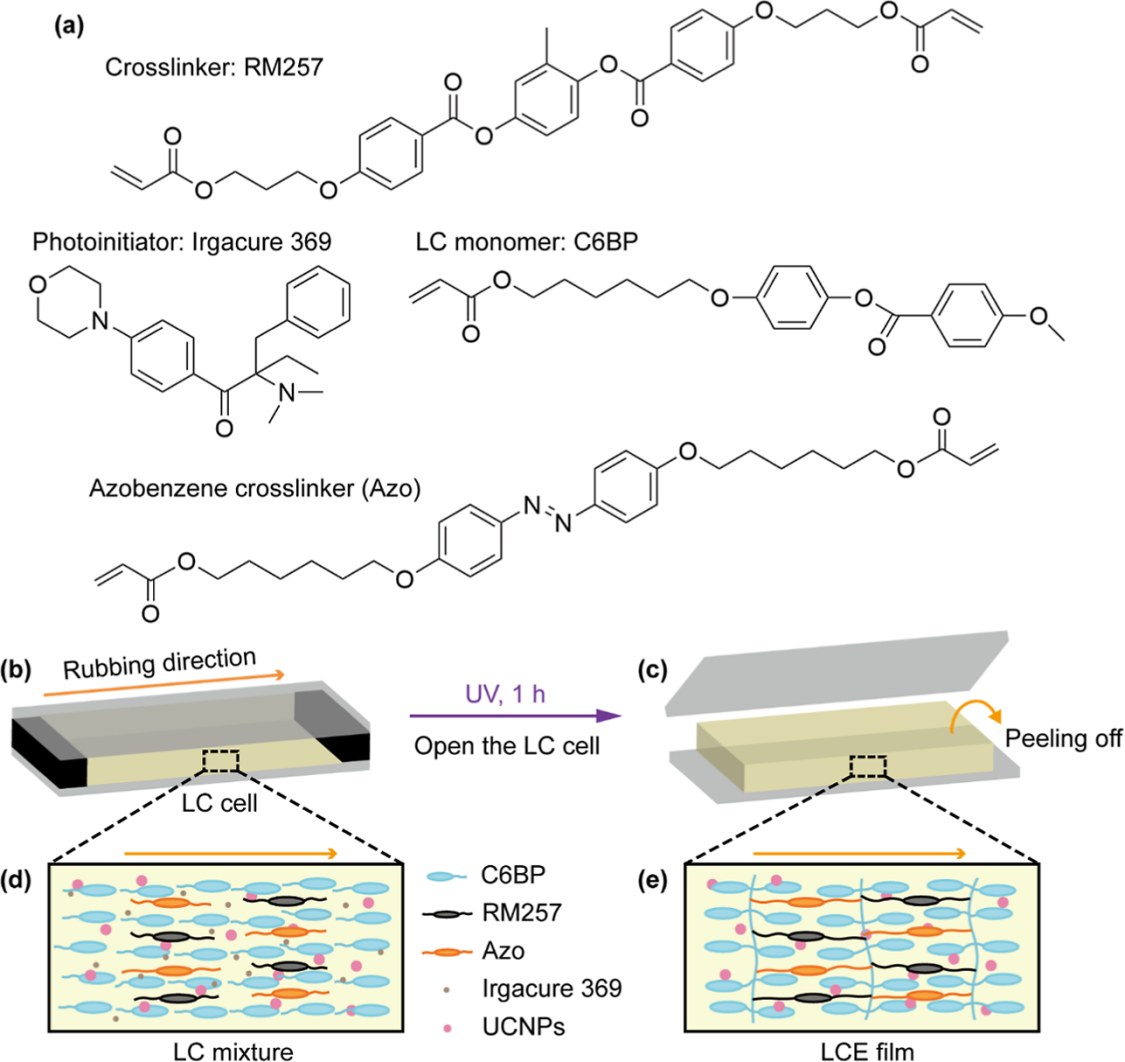
Fabrication of the UCNPs/Azo-LCE film. (a) Chemical structures of the components used in the LCE system: cross-linker RM257, photoinitiator Irgacure 369, monoacrylate LC monomer C6BP, and azobenzene cross-linker (Azo). (b) Schematic illustration of the LC cell with parallel rubbing alignment layers. The LC mixture was filled into the cell. (c) After UV irradiation (6 W, 365 nm, 1 h), the cell was opened, and the freestanding LCE film was obtained by peeling it off from the substrate. (d,e) Magnified schematics showing the internal structure of the LC mixture before (d) and the cross-linked LCE film after (e) UV photopolymerization.

Microgroove-patterned LCE films were fabricated via a template-replication method. Prestructured aluminum alloy templates bearing periodic microgroove arrays were used directly as topographic molds without further modification. The groove patterns on the templates had a fixed groove depth of 20 μm, a fixed ridge width of 20 μm, and variable groove widths (*W*) of 10, 30, 50, 70, and 90 μm. The groove sidewalls exhibited a draft angle of approximately 15° from the vertical, inherent to the original template fabrication process (Figure S1). The groove-patterned UCNPs/Azo-LCE films were prepared following the same LC cell assembly except that one PI-coated glass substrate was replaced by the aluminum alloy template (groove side facing inward) to imprint the microgroove topography onto the cured film.

### 2.3 Photothermal Characterization

The photothermal response of the UCNPs/Azo-LCE composite film and the Azo-LCE film was evaluated using an 808 nm CW NIR laser. A thermal imaging camera (FLIR C3-X) recorded the surface temperature during irradiation at power densities of 4, 8, 16, and 24 W cm^–2^ for 20 s per exposure. Four independent measurements were performed at each power density, and the average temperature over the last 10 s of each 20 s cycle was taken as a single data point.

To assess photothermal effects under cell culture conditions, a 35 mm glass-bottom dish containing 2 mL of complete culture medium was placed on a 37 °C heating stage. The medium was irradiated at power densities of 1, 2, 3, 4, 6, and 8 W cm^–2^ (spot size: 0.9 × 1.2 cm^2^) for 10 min. Temperature at the dish center was recorded at 30 s intervals. Baseline temperature *T*_0_ was averaged from five readings over a 2 min preirradiation period. Steady-state temperature *T*_ss_ was averaged from five readings between 8 and 10 min of irradiation, and Δ*T* = *T*_ss_ – *T*_0_ was calculated. For on–off cycling, the 8 W cm^–2^ laser was operated in 20 s on/20 s off mode for 50 cycles, recording *T*_max_ and *T*_min_ per cycle.

### 2.4 Calcein AM/PI Staining for Cell Viability

H9c2 cells were seeded in 6-well plates onto flat UCNPs/Azo-LCE films, microgrooved UCNPs/Azo-LCE films with groove widths of 10, 30, 50, 70, and 90 μm, and glass coverslips at a density of 1.63 × 10^4^ cells cm^–2^ and cultured for 24 h. After removing the culture medium, cells were stained with a freshly prepared Calcein AM/propidium iodide (PI) working solution, prepared by diluting Calcein AM and PI 1000× stocks separately in the assay buffer at 1:1000. For each well, 1 mL of the working solution was added, followed by incubation at 37 °C for 30 min in the dark. Samples were then immediately imaged using a fluorescence microscope. Calcein AM (green fluorescence, E_*x*_/E_*m*_ = 494/517 nm) was used to label live cells, whereas PI (red fluorescence, E_*x*_/E_*m*_ = 535/617 nm) was used to label dead cells.

Three independent biological replicates were performed using H9c2 cells from three different frozen vials, with cells at passages 1–5. For each biological replicate, two technical replicates were prepared as two separate wells per condition. One randomly selected field containing at least 150 cells was analyzed for each technical replicate. The numbers of live and dead cells were counted, and cell viability was calculated as Live cells (%) = [green cells/(green cells + red cells)] × 100%. The mean of the two technical replicates was used as one biological replicate data point, and data are presented as mean ± SD (*n* = 3). Methanol-treated H9c2 cells on glass coverslips (70% methanol, 5 min) served as the dead-cell positive control, and untreated cells on glass coverslips served as the live-cell control. Background fluorescence was evaluated using cell-free flat UCNPs/Azo-LCE films and was negligible.

For statistical analysis, one-way ANOVA followed by Dunnett’s multiple comparisons test was performed. Two comparison sets were used: microgrooved UCNPs/Azo-LCE films with groove widths of 10, 30, 50, 70, and 90 μm versus the flat UCNPs/Azo-LCE film, and all UCNPs/Azo-LCE groups, including flat and microgrooved films, versus the glass coverslip control.

### 2.5 Immunofluorescence Staining

For immunofluorescence staining, the cultured cells on LCE substrates were first rinsed with PBS to remove residual medium. The cells were then fixed with 4% PFA at room temperature for 15 min, followed by three PBS washes (5 min each). Permeabilization was performed by incubating the samples with 0.1% Triton X-100 for 25 min at room temperature, followed by three PBS washes (5 min each). To minimize nonspecific binding, the samples were blocked with 1% BSA solution for 30 min. After removing the blocking solution, F-actin was stained with Alexa Fluor 488 phalloidin at room temperature in the dark for 30 min. The samples were then washed three times with PBS (5 min each) and mounted with DAPI-containing mounting medium for nuclear visualization. The mounted samples were stored in the dark for 24 h before imaging. Fluorescence images of the cytoskeletal architecture and nuclear morphology were acquired using a Nikon Inverted Ti2-A Microscopy wide-field epifluorescence imaging system equipped with filter sets for DAPI (excitation ∼360 nm) and Alexa Fluor 488 (excitation ∼488 nm).

### 2.6 Image Analysis and Quantification of Cell Alignment

Fluorescence images were analyzed using ImageJ software. Cells were quantified from randomly selected fields of view for each substrate condition. The orientation angle (α) of each cell was defined as the angle between the major axis of the cell body and the groove direction, which was taken as the positive *y*-axis, with values ranging from −90° to 90°. Angular distribution histograms were generated from the measured cell orientation angles, and the percentage of cells in each angular bin was calculated as the number of cells within that bin divided by the total number of analyzed cells.

For morphometric analysis, three independent biological replicates were performed using H9c2 cells from three different frozen vials, and cells at passages 1–5 were used. In each biological replicate, two technical replicates were prepared. For each technical replicate, one randomly selected field of view containing at least 150 cells per condition was analyzed. The mean value of the two technical replicates was taken as one data point for that biological replicate. Final data are presented as mean ± SD of the three biological replicate means (*n* = 3).

The nematic order parameter *S* was calculated to quantify the degree of cell alignment

$$S=2\left\langle \cos^{2}\alpha \right\rangle -1$$

where the angle brackets denote averaging over all measured cells. An *S* value of 1 indicates perfect alignment parallel to the groove direction, 0 corresponds to random orientation, and −1 represents perpendicular alignment. The cell aspect ratio (AR) was determined as the ratio of the major axis length to the minor axis length of the best-fit ellipse for each cell. The projected cell spreading area was measured from the thresholded F-actin fluorescence signal of individual cells. Cell density was calculated as the number of DAPI-stained nuclei within a field of view divided by the field area. For nuclei touching the image boundaries, only those intersecting the left and upper borders were counted. Summary statistics are reported as mean ± SD unless otherwise specified. Statistical analysis was performed using GraphPad Prism. Differences among substrate conditions were evaluated by one-way analysis of variance (ANOVA) followed by Dunnett’s multiple comparisons test, with the flat LCE substrate serving as the control group. A *p* value less than 0.05 was considered statistically significant.

## 3 Results and Discussion

### 3.1 Synthesis and Characterization of UCNPs

The CSS-UCNPs were synthesized via a high-temperature coprecipitation method following established protocols. The structural design of the CSS-UCNPs is illustrated in Figure S2. The morphology, crystal structure, and optical properties of the resulting nanoparticles are summarized in Figure 2. TEM analysis (Figure 2a) revealed nanoparticles with a predominantly hexagonal morphology and an average diameter of 55.7 ± 5.3 nm (Figure 2b). The XRD patterns of all three synthetic stages (C-UCNPs, CS-UCNPs, and CSS-UCNPs) were well indexed to the standard hexagonal-phase β-NaYF_4_ reference (PDF#16-0334) (Figure S3), confirming that the pure hexagonal crystal structure was preserved throughout the successive shell growth steps without phase transformation. The progressive increase in nanoparticle diameter from 24.3 ± 2.2 nm (C-UCNPs) to 41.5 ± 3.5 nm (CS-UCNPs) and finally 55.7 ± 5.3 nm (CSS-UCNPs), as revealed by TEM analysis of each synthetic stage (Figure S4, Table S1), confirmed the successful epitaxial growth of the Yb/Nd active shell (∼8.6 nm per side) and the inert NaYF_4_ passivation shell (∼7.1 nm per side).

**Figure 2:**
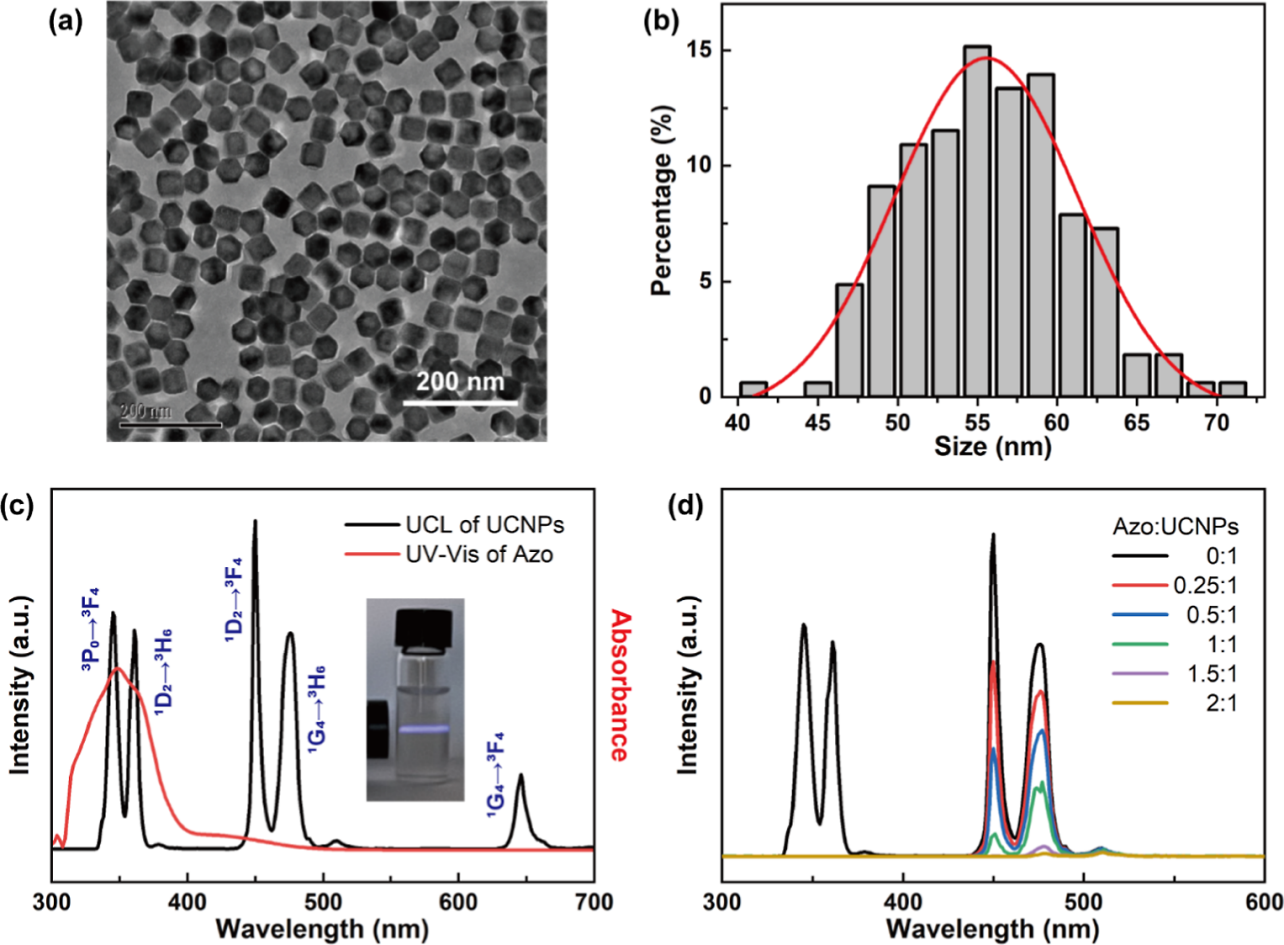
(a) TEM image of CSS-UCNPs. (b) Size distribution histogram of CSS-UCNPs obtained from TEM image analysis. (c) UCL spectrum of CSS-UCNPs (0.5 mg mL^–1^ in cyclohexane) and UV–vis absorption spectrum of Azo (0.5 mg mL^–1^ in acetone); the inset shows a digital photograph of CSS-UCNPs emitting UV light under 808 nm CW NIR irradiation (600 mW). (d) UCL spectra of Azo/UCNPs with different Azo-to-UCNPs ratios (fixed UCNPs concentration 0.5 mg mL^–1^ in acetone).

Under 808 nm CW NIR laser excitation, the CSS-UCNPs exhibited characteristic Tm^3+^ UCL peaks at 345 nm (^3^P_0_→^3^F_4_), 361 nm (^1^D_2_→^3^H_6_), 451 nm (^1^D_2_→^3^F_4_), 476 nm (^1^G_4_→^3^H_6_), and 647 nm (^1^G_4_→^3^F_4_) (Figure 2c). Notably, the UV emission bands and part of the blue emission region show substantial spectral overlap with the absorption band of the azobenzene derivative (Figure 2c). Furthermore, UV–vis spectroscopy confirmed the rapid and reversible photoisomerization of Azo: under 365 nm UV irradiation (20 W), the π→π* absorption band of the *trans*-isomer (∼350 nm) decreases progressively while the *n*→π* band of the *cis*-isomer (∼440 nm) increases slightly, reaching a photostationary state within 20 s. Subsequent 450 nm blue light irradiation (10 W) almost fully recovers the original absorption spectrum within 20 s, indicating nearly complete *cis*-to-*trans* back-isomerization (Figure S5). The combination of spectral overlap and efficient photoswitching at these wavelengths establishes the basis for using 808 nm NIR excitation to indirectly drive the bidirectional photoisomerization of azobenzene chromophores via UCL.

To further examine the optical interaction between UCNP emission and azobenzene absorption, the UCL spectra of Azo/UCNPs mixtures with different Azo-to-UCNPs mass ratios were measured, as shown in Figure 2d. Compared with the spectrum of pure CSS-UCNPs, the addition of Azo leads to pronounced quenching of the UV emission bands at 345 and 361 nm. At an Azo/UCNPs ratio of 1:0.25, these UV peaks are nearly undetectable, indicating that the UV photons emitted by the UCNPs can be effectively absorbed by the azobenzene groups. As the Azo proportion is further increased, the blue emission bands at 451 and 476 nm also gradually decrease, suggesting enhanced attenuation of the shorter-wavelength visible emission. These spectral changes are consistent with the overlap between the UCNP emission and Azo absorption shown in Figure 2c, and provide direct evidence that the emitted UV and part of the blue photons from the UCNPs can be efficiently harvested by the azobenzene component.

### 3.2 Fabrication and Characterization of UCNPs/Azo-LCE Films

The fabrication process of UCNPs/Azo-LCE films is schematically illustrated in Figure 1. The Azo-LCE films were prepared with a monomer-to-cross-linker molar ratio of C6BP/RM257/Azo/Irgacure 369 = 89:9:1:1 and a cell spacer thickness of 30 μm. This optimized formulation and geometry were therefore adopted for all subsequent experiments, yielding films with a measured thickness of 34.12 ± 1.51 μm. To introduce NIR responsiveness, UCNPs were incorporated into the optimized Azo/LCE matrix at varying UCNPs/Azo weight ratios (0.25:1, 0.5:1, 1:1, 1.5:1, and 2:1). The optimal UCNPs loading was determined to be UCNPs/Azo = 0.5:1 (w/w), based on a combination of spectroscopic, thermal, and photomechanical results. In the UCL measurements shown in Figure 2d, this composition corresponds to an Azo/UCNPs ratio of 2:1. At this ratio, the UV emission of the UCNPs is effectively quenched by Azo absorption, indicating efficient optical coupling between the two components. DSC analysis of the LC precursor mixtures further supported this selection: the 0.5:1 formulation exhibited a single sharp nematic-to-isotropic transition at 47.3 °C, comparable to that of the neat Azo/LCE mixture (50.2 °C), whereas a higher loading of 1:1 produced two endothermic peaks indicative of phase separation (Figure S6). POM observation of the cured films under crossed polarizers confirmed the presence of birefringence, indicating the retention of liquid crystalline order after photopolymerization for UCNPs/Azo-LCE films (Figure S7).

Photomechanical testing under 16 W cm^–2^ NIR irradiation revealed that among all tested UCNPs loadings, only the 0.5:1 formulation produced the most pronounced and reproducible macroscopic bending deformation. In all, these results indicate that this composition provides a suitable balance between efficient utilization of the upconverted UV photons and preservation of the liquid-crystalline order required for anisotropic photomechanical actuation. This narrow performance window can be rationalized by considering two competing effects: at lower UCNPs loadings, the upconversion luminescence intensity is insufficient to drive azobenzene photoisomerization above the threshold required for macroscopic actuation; at higher loadings, the pronounced *T*_NI_ depression severely compromises the nematic order parameter, thereby diminishing the anisotropic strain that constitutes the driving force for photomechanical deformation. Unless otherwise specified, all subsequent experiments were conducted using UCNPs/Azo-LCE films prepared under these optimized conditions.

### 3.3 NIR-Driven Photomechanical Actuation Mechanism

The actuation mechanism and photomechanical behavior of UCNPs/Azo-LCE films under 808 nm NIR irradiation are illustrated in Figure 3. As shown schematically in Figure 3a,b, the initially flat LCE film undergoes bending deformation toward the light source upon NIR exposure. The molecular-level origin of this response is depicted in Figure 3c,d,i. Upon 808 nm irradiation, the embedded UCNPs convert NIR photons into UV/blue upconversion luminescence (∼345, 361, 451, 476 nm) via a multiphoton energy transfer process (Figure 3i). To further elucidate the optical characteristics of the UCNPs/Azo system, the upconversion luminescence spectra of samples with different UCNPs/Azo ratios were recorded under identical excitation conditions (Figure S8). The 0:1 sample showed no detectable UCL signal, while characteristic UV and blue emission bands appeared after incorporation of UCNPs and generally increased with increasing UCNP content, indicating a composition-dependent optical output of the hybrid system. Notably, the enhancement became less pronounced at higher UCNPs/Azo ratios, suggesting that the emission increase was not strictly linear with UCNP loading. The locally generated UV photons are absorbed by neighboring azobenzene chromophores, triggering *trans*-to-*cis* photoisomerization that disrupts the local nematic order (Figure 3c,d). Because the upconverted luminescence is strongly reabsorbed by surrounding azobenzene units within the Azo cross-linker, its intensity is expected to establish a rapid decay with increasing depth into the film, following a Beer–Lambert-type attenuation profile.^51^ This establishes a pronounced through-thickness gradient in the *cis*-isomer population: the irradiated surface accumulates a high *cis*-fraction with substantially reduced nematic order, while the bulk retains predominantly *trans*-isomers with intact mesogenic alignment. The resulting asymmetric order parameter gradient produces anisotropic contraction on the irradiated side, generating a bending moment that drives the film to curl toward the NIR light source along the rubbing direction. The bending angle (θ) was defined as the angle between the horizontal baseline and the line connecting the midpoint of the fixed end and the midpoint of the free end of the bent film (Figure 3e). Digital photographs of a representative UCNPs/Azo-LCE film in the initial flat state (Figure 3f), the bent state under 808 nm NIR irradiation (Figure 3g), and the recovered state after removal of the light (Figure 3h) confirm the reversible bending behavior. Upon NIR exposure, the film exhibited clear bending toward the light source; after the light was switched off, the film partially recovered its original flat shape but retained a residual deformation, which is attributed to incomplete *cis*-to-*trans* back-isomerization of azobenzene units within the limited recovery period. A representative video of the reversible bending-recovery process is provided in Movie S1.

**Figure 3:**
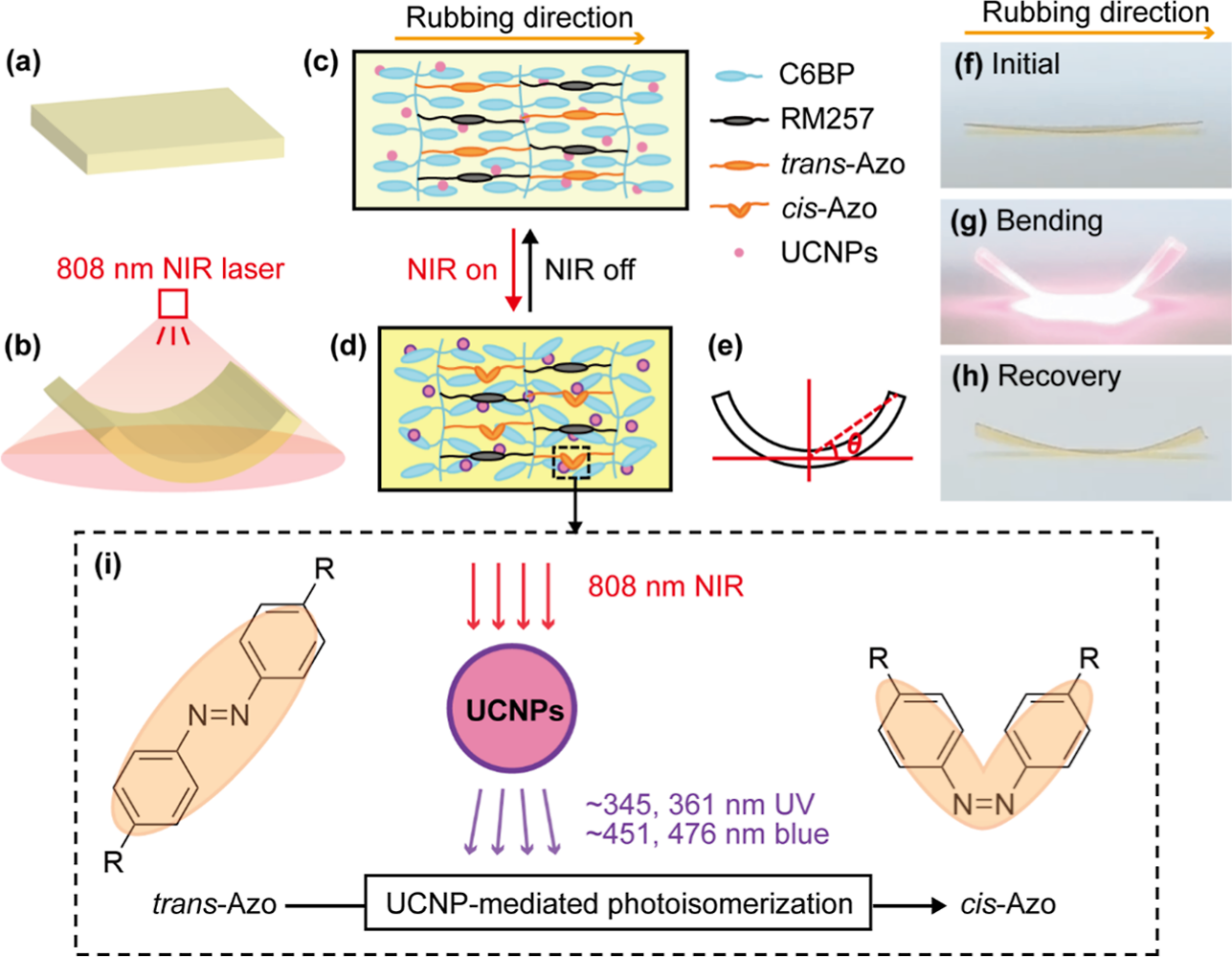
NIR-light-driven bending behavior and mechanism of the UCNPs/Azo-LCE film. (a,b) Schematic illustration of the LCE film in (a) the initial flat state and (b) the bending state under 808 nm NIR laser irradiation. (c,d) Magnified schematics showing the internal molecular structure of the LCE film (c) before (NIR off) and (d) during (NIR on) NIR irradiation, illustrating the *trans*-to-*cis* isomerization of azobenzene cross-linkers and the resulting disruption of molecular order. (e) Definition of the bending angle θ. (f–h) Digital photographs of a representative UCNPs/Azo-LCE film at the (f) initial, (g) bending, and (h) recovery states. (i) Schematic of the UCNP-mediated azobenzene photoisomerization pathway. The film dimension is 10 mm × 2 mm × 34.12 ± 1.51 μm, and the NIR irradiation spot size is 4.8 mm × 5.2 mm.

This mechanism was verified by control experiments: an Azo-LCE film without UCNPs showed no detectable bending under identical 808 nm NIR irradiation (16 W cm^–2^), yet bent readily upon direct 365 nm UV exposure, confirming that UCNPs are necessary to convert NIR photons into UV wavelengths required to trigger azobenzene photoisomerization (Figure S9). To further exclude the possibility of photothermal-induced deformation, we monitored the surface temperatures of both films under 808 nm NIR irradiation at various power densities. The two films exhibited nearly identical heating profiles across the entire range (4–24 W cm^–2^), indicating that the incorporation of UCNPs did not introduce a detectable additional photothermal contribution under the tested conditions (Figure S10a). At the power densities used for cyclic actuation and cell experiments (4–16 W cm^–2^), the surface temperature remained below the *T*_NI_ of the composite (∼47 °C, Figure S6). Moreover, the Azo-LCE film without UCNPs exhibited a similar temperature rise but did not bend (Figures S9 and S10a; Table S2), confirming that heating alone is insufficient to induce the observed deformation. Instead, the UCNPs convert 808 nm NIR light into UV/blue upconversion emission, which triggers azobenzene photoisomerization and thereby drives the photomechanical response. As only the UCNPs/Azo-LCE film exhibited bending, this actuation was primarily attributed to UCNP-mediated azobenzene photoisomerization rather than the photothermal effect.

### 3.4 Power-Dependent Photomechanical Response and Cycling Durability

The power dependence of the photomechanical response was further investigated across the 4–24 W cm^–2^ range (Figure 4). The time-resolved bending angle profile recorded during a single bending–recovery cycle at each power density is shown in Figure 4a. The time required to reach θ_max_ depended on the power density: the peak typically occurred at approximately 5 s at 4 and 8 W cm^–2^, approximately 10 s at 16 W cm^–2^, and approximately 15 s at 24 W cm^–2^, as shown in Figure 4a. Although some sample-to-sample variation was observed, the maximum was always reached within the 20 s irradiation window under all tested conditions. θ_max_ after 20 s of NIR exposure systematically increased from 11.0 ± 0.3° (4 W cm^–2^), 14.1 ± 3.3° (8 W cm^–2^), 26.3 ± 1.8° (16 W cm^–2^) to 42.8 ± 2.6° (24 W cm^–2^) (Figure 4b). The increase in bending angle with power density is consistent with more effective UCNP-mediated photoisomerization of azobenzene units within the Azo cross-linker under stronger NIR irradiation. After NIR irradiation at 24 W cm^–2^, optical microscopy revealed no surface cracking, pitting, or delamination on the film. At this power density, the films exhibited reproducible bending over repeated irradiation cycles. After overnight relaxation, they fully recovered to the flat state and could be subjected to further repeated bending, indicating the absence of irreversible damage under the tested conditions. The cycling durability was further evaluated under periodic 808 nm NIR irradiation (16 W cm^–2^, 20 s on/20 s off) over 50 consecutive cycles (Figure 4c). Movie S1 presents an accelerated representative clip of the first five bending–recovery cycles recorded (16 W cm^–2^, 8× playback speed). Throughout the test, the bent state exhibited a mean θ_max_ of 25.15 ± 1.41° and the recovered state maintained a mean θ_min_ of 12.54 ± 0.99°, with cycle-to-cycle coefficients of variation of 5.60% and 7.89%, respectively. A slight upward drift in both θ_max_ and θ_min_ was observed over the course of cycling, which may be attributed to the gradual accumulation of residual *cis*-azobenzene units that do not fully revert to the *trans*-state within each 20 s recovery period. Nevertheless, the actuation amplitude (θ_max_–θ_min_) remained stable throughout the 50 cycles, demonstrating reliable and repeatable photomechanical performance of UCNPs/Azo-LCE films under prolonged periodic NIR stimulation.

**Figure 4:**
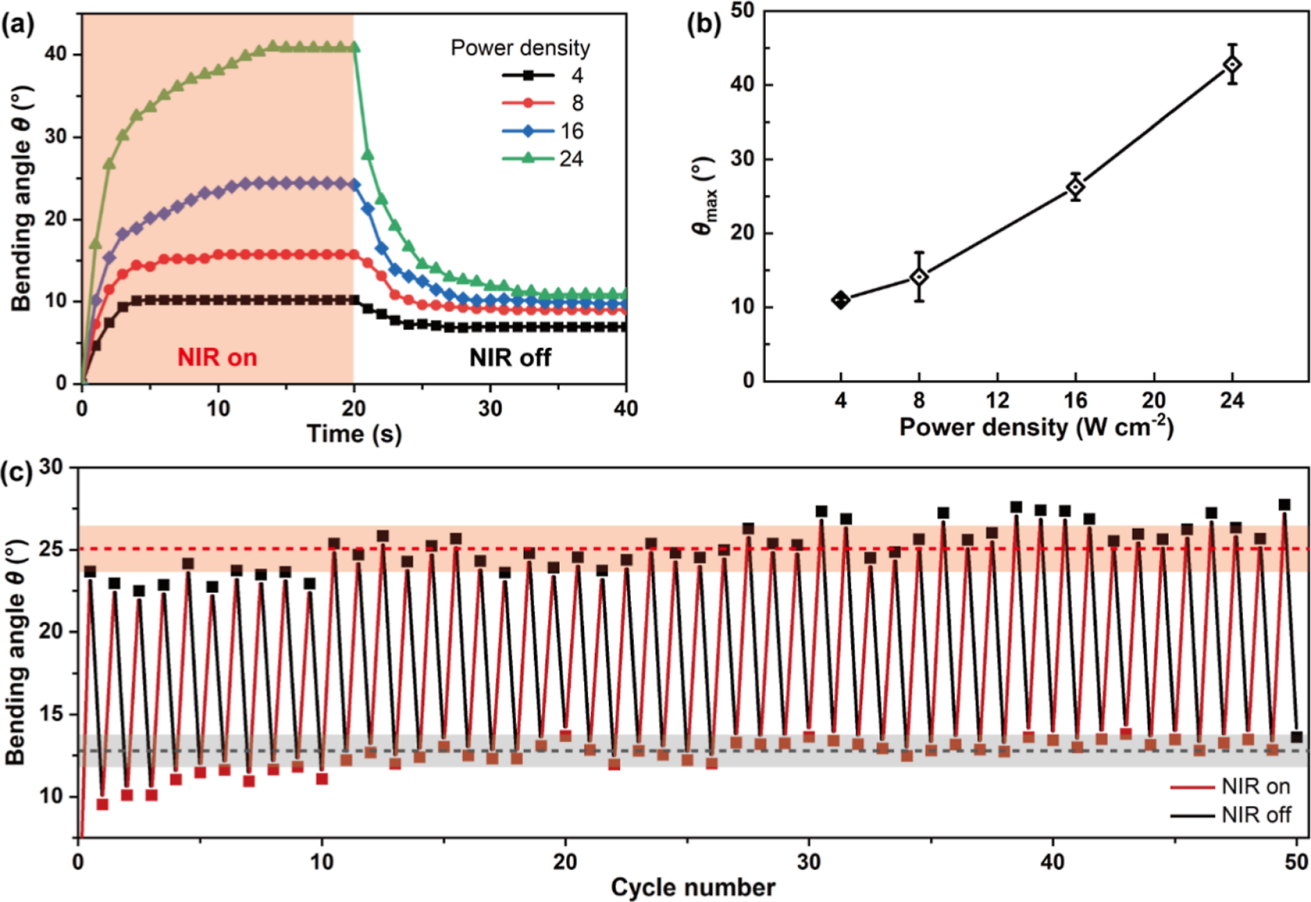
Power-dependent photomechanical response of UCNPs/Azo-LCE films under 808 nm NIR irradiation. (a) Time-resolved bending angle profiles during a single bending-recovery cycle at various power densities (4, 8, 16, and 24 W cm^–2^); the light was turned on at *t* = 0 s and off at *t* = 20 s. (b) Maximum bending angle (θ_max_) as a function of power density after 20 s of 808 nm NIR exposure. Error bars represent standard deviations (*n* = 4). (c) Cycling durability of a UCNPs/Azo-LCE film under periodic 808 nm NIR irradiation (16 W cm^–2^, 20 s on/20 s off, 50 cycles), showing the bending angles recorded at NIR-on (red line) and NIR-off (black line) states in each cycle. The red and gray dashed lines represent the mean values of θ_max_ and θ_min_ over 50 cycles, respectively; the corresponding shaded bands indicate ± one standard deviation.

The photomechanical results above demonstrate that the UCNPs/Azo-LCE actuator can produce bending angles spanning from ∼11° to ∼43° across the 4–24 W cm^–2^ range and sustain stable cyclic operation at 16 W cm^–2^. For cell culture applications, however, the full actuation amplitude is not required; modest, reproducible surface deformations at lower power densities are sufficient to deliver physiologically relevant mechanical cues to adherent cells. Operating at reduced power densities also minimizes the photothermal burden on the culture environment—a critical consideration given that even a few degrees of sustained heating can alter cellular metabolism. We therefore identified 8 W cm^–2^ as the target operating condition for biologically relevant actuation: it lies above the onset threshold for detectable bending (4 W cm^–2^), yet is expected to produce substantially less heating than the 16–24 W cm^–2^ regime used for materials-level characterization. To validate this rationale, a systematic photothermal assessment was performed at power densities of 1–8 W cm^–2^ under cell-culture-mimicking conditions.

### 3.5 Photothermal Assessment of NIR Irradiation

Although the photoisomerization-based actuation mechanism does not inherently require a temperature rise, any residual photothermal effect arising from NIR absorption by the aqueous culture medium must be assessed to ensure biological compatibility. To this end, the temperature of complete culture medium was monitored in real time by infrared thermal imaging during 808 nm CW laser exposure at power densities ranging from 1 to 8 W cm^–2^ using an elliptical laser spot size of 0.9 × 1.2 cm^2^ (Figure 5a). This spot size was larger than that used for the actuation experiments (rectangle, 4.8 × 5.2 mm^2^) to better mimic the cell culture configuration. Although spot size may influence heat dissipation, the power density was kept constant, and heat spreading in the aqueous medium helps reduce local temperature gradients; therefore, the measured temperature rise provides a reasonable estimate of the thermal environment during cell illumination. Representative infrared thermal images at each power density are shown in Figure S11. Infrared thermal imaging further indicated that the temperature increase was largely confined to the directly irradiated region, whereas the surrounding medium remained close to the baseline temperature, suggesting limited lateral heat propagation under the present conditions.

**Figure 5:**
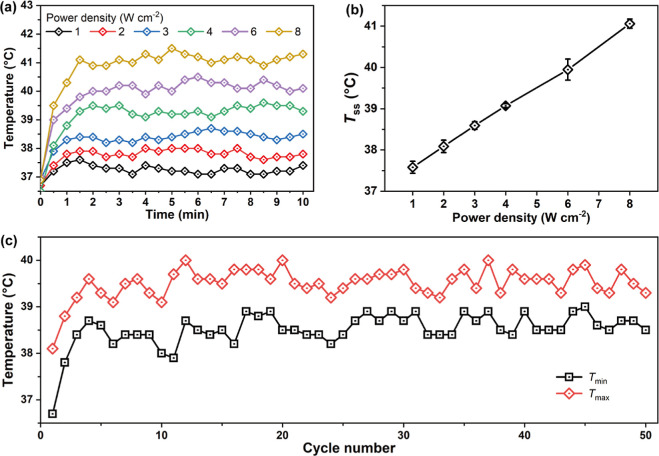
Photothermal characterization of 808 nm NIR irradiation in a cell-culture-mimicking environment. (a) Real-time temperature profiles of culture medium (2 mL, 35 mm dish, baseline 37 °C) under 808 nm CW irradiation at power densities of 1, 2, 3, 4, 6, and 8 W cm^–2^. (b) Steady-state temperature (*T*_ss_) as a function of power density; error bars represent mean ± SD from three independent measurements. The dashed line is a linear fit. (c) Maximum (*T*_max_) and minimum (*T*_min_) temperatures recorded during 50 consecutive on–off irradiation cycles (20 s on/20 s off) at 8 W cm^–2^, demonstrating the absence of cumulative thermal buildup over extended cyclic operation.

Upon irradiation, the medium temperature rose rapidly within the first ∼2 min and then plateaued, reaching a power-density-dependent steady-state temperature (*T*_ss_). As shown in Figure 5b, *T*_ss_ increased approximately linearly with power density, from 37.07 ± 0.09 °C at 1 W cm^–2^ to 41.06 ± 0.05 °C at 8 W cm^–2^, corresponding to temperature elevations of 0.26 and 4.36 °C, respectively. Even at the highest tested power density, the steady-state temperature remained within a range often considered compatible with short-term mammalian cell exposure.^52^ At intermediate power densities relevant to the photomechanical actuation demonstrated above (4–8 W cm^–2^), Δ*T* was limited to 2.29–4.36 °C, confirming that 808 nm irradiation at the power densities employed in this study introduces only a modest thermal perturbation to the culture environment. To further assess the local temperature at the film surface, a supplementary floating-film experiment was performed (Figure S12). Although this configuration does not fully replicate the actual submerged cell-culture geometry, the measured film-surface temperature under 8 W cm^–2^ irradiation was comparable in magnitude to that observed in the cell-culture-mimicking photothermal assessment, suggesting that no pronounced additional local overheating occurred under the tested condition.

To further evaluate whether cyclic NIR irradiation leads to cumulative thermal buildup, on–off cycling experiments were performed at 8 W cm^–2^ (20 s on/20 s off, 50 cycles). As shown in Figure 5c, after an initial equilibration period of approximately 4 cycles, both *T*_max_ and *T*_min_ reached steady oscillation. Over the subsequent 46 cycles, *T*_max_ = 39.53 ± 0.28 °C and *T*_min_ = 38.57 ± 0.25 °C (mean ± SD, *n* = 46), exhibiting no progressive drift or thermal ratcheting. Importantly, the *T*_max_ values recorded during cyclic irradiation at 8 W cm^–2^ remained consistently below 40 °C, which is lower than the ∼41 °C *T*_ss_ observed under continuous irradiation at the same power density. This difference arises because the 20 s irradiation period is insufficient for the medium to reach thermal equilibrium, and the intervening 20 s off-period allows effective heat dissipation. Therefore, under practically relevant pulsed operation conditions, the thermal burden imposed on cultured cells is expected to be even milder than what the continuous-irradiation data suggest.

Taken together, these results demonstrate that 808 nm NIR irradiation at the power densities and duty cycles employed in this study produces only modest and reversible heating in aqueous culture environments under the tested conditions, supporting the thermal compatibility of the UCNPs/Azo-LCE actuator system with cell culture applications.

### 3.6 Quantitative Cell Viability on UCNPs/Azo-LCE Substrates

To evaluate the potential of UCNPs/Azo-LCE films as topographically engineered cell culture substrates, microgroove-patterned LCE films with groove widths of 10, 30, 50, 70, and 90 μm, along with a flat nonpatterned LCE film, were fabricated as individual 5 × 5 mm pieces and co-mounted onto a single 25 × 25 mm glass coverslip. The surface topography of the grooved LCE substrates was confirmed by scanning electron microscopy (SEM). Top-view images showed well-defined, periodic grooves with widths of 10, 30, 50, 70, and 90 μm (Figure S13a–e), and a cross-sectional image revealed a trapezoidal groove profile with a sidewall angle (Figure S13k), consistent with the anisotropic etching geometry of the alumina template and confirming faithful pattern transfer to the LCE surface. The remaining exposed glass surface served as a built-in material control. This single-coverslip design ensured that all seven conditions, including five groove widths, a flat LCE topographic control, and the bare glass reference, were cultured and stained simultaneously in a single procedure, thereby eliminating intersample processing variability. As a commonly used in vitro cardiomyoblast model, H9c2 cells were selected to evaluate the cellular compatibility and topographic guidance effect of the substrates.

To further evaluate the short-term cytocompatibility of the UCNPs/Azo-LCE substrates, Calcein AM/PI live/dead staining was performed after 24 h of H9c2 cell culture. Microgrooved UCNPs/Azo-LCE films with groove widths of 10, 30, 50, 70, and 90 μm, a flat UCNPs/Azo-LCE film, and glass coverslips were examined in parallel. Representative merged fluorescence images for the *W* = 50 μm microgrooved UCNPs/Azo-LCE film and the flat UCNPs/Azo-LCE film are shown in Figure 6a, while the complete set of fluorescence images for all substrate conditions is provided in Figure S14.

**Figure 6:**
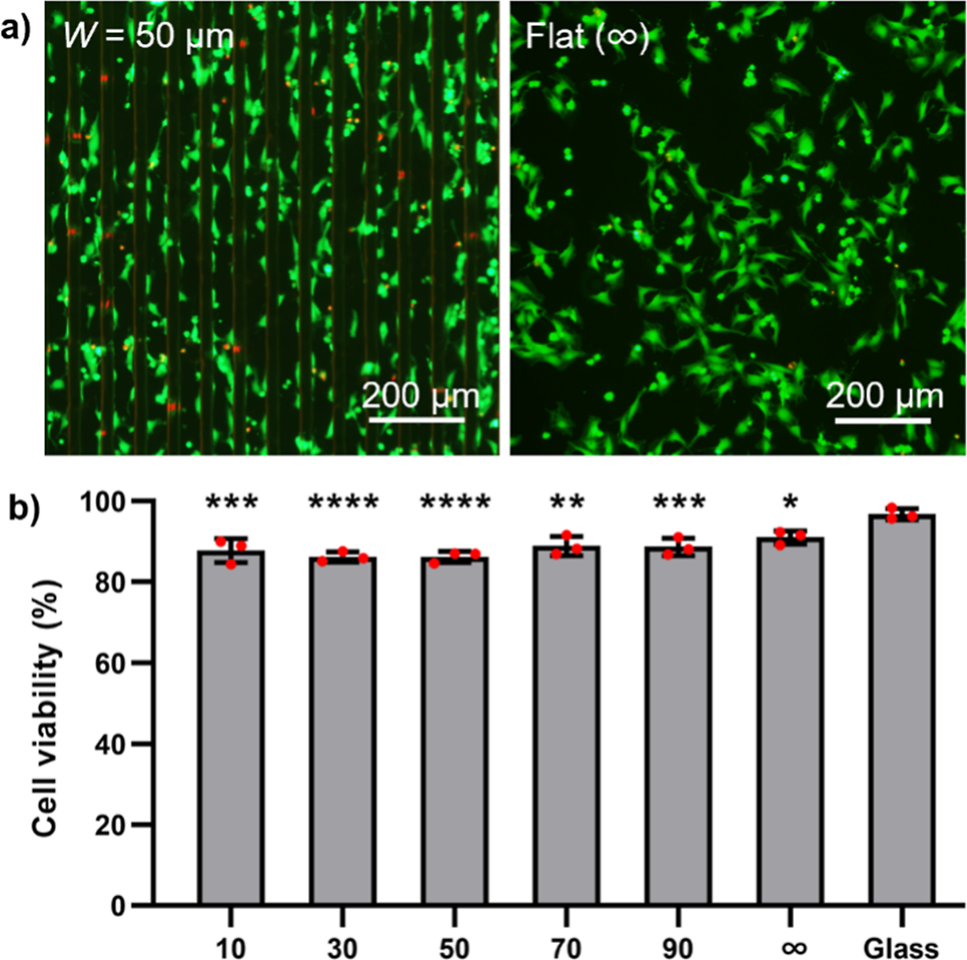
Quantitative analysis and representative live/dead staining images of H9c2 cells cultured on UCNPs/Azo-LCE substrates for 24 h. (a) Representative merged Calcein AM/PI fluorescence images of H9c2 cells cultured on a microgrooved UCNPs/Azo-LCE film with *W* = 50 μm and a flat UCNPs/Azo-LCE film. Live cells are shown in green and dead cells in red. Scale bar: 200 μm. (b) Quantitative cell viability of H9c2 cells cultured on microgrooved UCNPs/Azo-LCE films with groove widths of 10, 30, 50, 70, and 90 μm, the flat UCNPs/Azo-LCE film (*W* = ∞), and glass coverslips. Data are presented as mean ± SD (*n* = 3). No significant differences were detected between the microgrooved groups and the flat UCNPs/Azo-LCE film. Significance markers indicate comparisons with the glass coverslip control. *p < 0.05, **p < 0.01, ***p < 0.001, ****p < 0.0001.

In all groups, the majority of cells exhibited strong green fluorescence, whereas only a small number of PI-positive dead cells were observed. Quantitative analysis (Figure 6b) showed that the cell viability was 87.77 ± 2.95%, 85.32 ± 2.55%, 86.15 ± 1.37%, 88.88 ± 2.42%, and 88.68 ± 2.14% on microgrooved UCNPs/Azo-LCE substrates with groove widths of 10, 30, 50, 70, and 90 μm, respectively. The viability on the flat UCNPs/Azo-LCE film was 91.02 ± 1.64%, while that on the glass coverslip control was 96.78 ± 1.38%. All UCNPs/Azo-LCE groups maintained cell viability above 85%, indicating good short-term cytocompatibility under the present culture conditions.

Statistical analysis showed that the cell viabilities on the microgrooved UCNPs/Azo-LCE substrates were not significantly different from that on the flat UCNPs/Azo-LCE film. This result indicates that the introduction of microgroove topography did not induce statistically significant additional cytotoxicity relative to the unpatterned LCE substrate. In contrast, compared with the glass coverslip control, all UCNPs/Azo-LCE substrate groups showed significantly lower cell viability, with significance levels of *p* < 0.001, *p* < 0.0001, *p* < 0.0001, *p* < 0.01, *p* < 0.001, and *p* < 0.05 for the 10, 30, 50, 70, 90 μm, and flat LCE groups, respectively.

The slightly lower viability observed on LCE-based substrates compared with glass may be related to differences in intrinsic material and surface properties of the cross-linked LCE matrix, which can influence protein adsorption and initial cell–substrate interactions. Importantly, because none of the microgrooved groups showed a significant reduction in viability compared with the flat LCE film, the decrease relative to glass is more likely attributable to the LCE substrate material itself rather than the microgroove topography. Taken together, these live/dead staining results demonstrate that the UCNPs/Azo-LCE substrates support good short-term H9c2 cell viability and that the microgrooved structures do not introduce additional cytotoxicity under the tested conditions.

### 3.7 Effect of Microgroove Topography on Cell Alignment

After confirming the short-term cytocompatibility of the UCNPs/Azo-LCE substrates, the same substrate configuration was further used to evaluate the effect of microgroove topography on H9c2 cell alignment. H9c2 cells were seeded and cultured for 24 h, after which cells were fixed and stained with Alexa Fluor 488 phalloidin for F-actin visualization and DAPI for nuclear identification. The resulting fluorescence micrographs and cell orientation angle distributions are presented in Figure 7, while the quantitative morphometric analyses are summarized in Figure 8.

**Figure 7:**
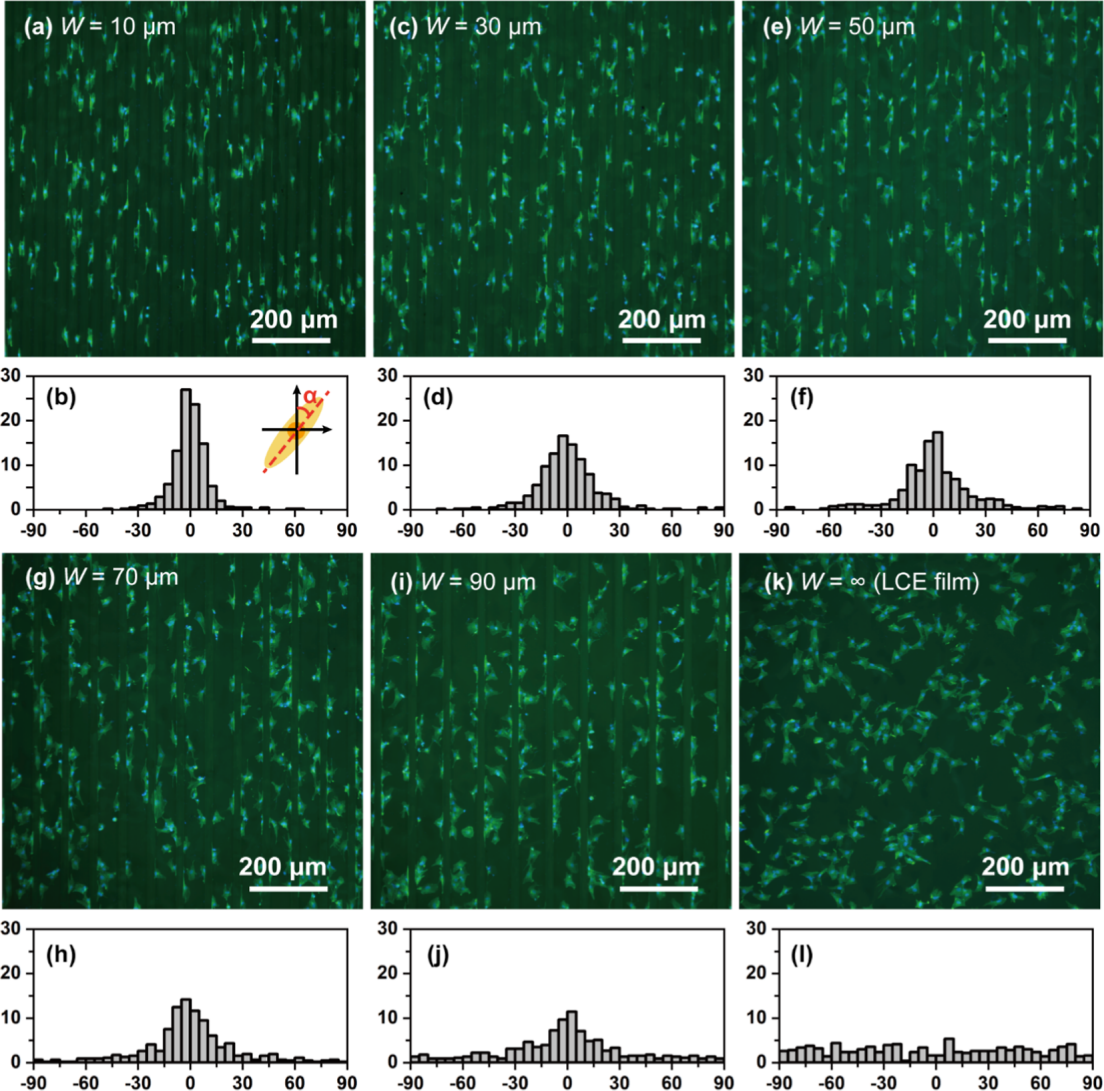
Fluorescence microscopy images and cell orientation distributions on grooved UCNPs/Azo-LCE substrates. Representative fluorescence images of H9c2 cells cultured for 24 h on LCE substrates with groove widths of (a) *W* = 10 μm, (c) 30 μm, (e) 50 μm, (g) 70 μm, (i) 90 μm, and (k) flat LCE film. F-actin is stained with Alexa Fluor 488 phalloidin (green) and nuclei with DAPI (blue). Scale bars: 200 μm. The corresponding angular distribution histograms are shown in (b), (d), (f), (h), (j), and (l). The inset in (b) schematically illustrates the definition of the orientation angle α relative to the groove direction.

**Figure 8:**
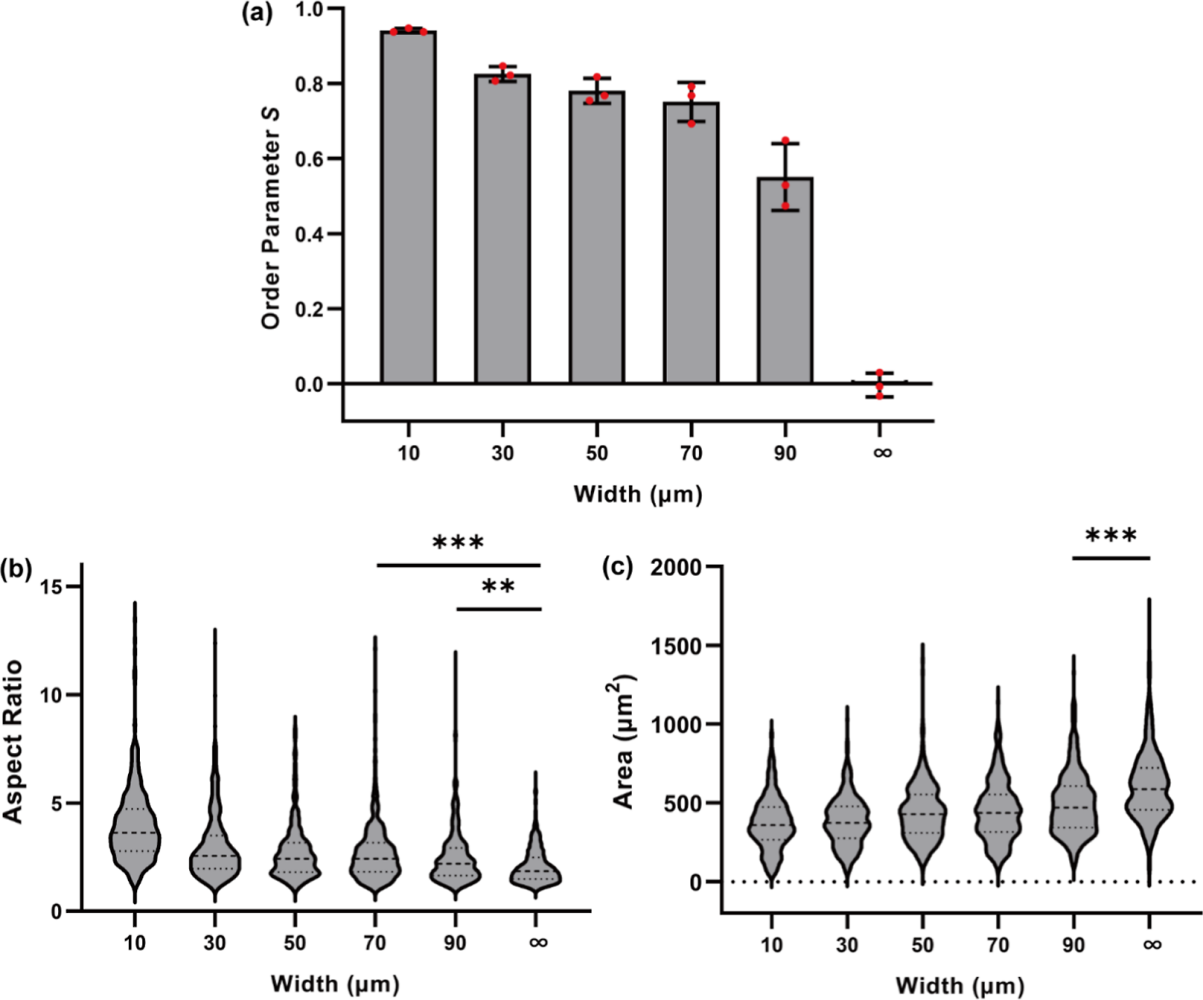
Quantitative analysis of H9c2 cell behavior on grooved LCE substrates with varying groove widths (*W* = 10, 30, 50, 70, and 90 μm) and a flat LCE control. (a) Nematic order parameter (*S*). (b) Cell aspect ratio (AR). (c) Projected cell area. Data in (a) are presented as mean ± SD from three independent experiments; data in (b,c) are violin plots of pooled single-cell measurements. Statistical analysis was performed using replicate means (*n* = 3) by one-way ANOVA followed by Dunnett’s multiple comparisons test versus the flat LCE control. For clarity, comparisons with *p* < 0.0001 are not annotated. **p < 0.01, ***p < 0.001.

Figure 7 presents representative fluorescence micrographs and corresponding orientation-angle histograms for cells cultured on the six LCE surfaces, including W = 10 μm (Figure 7a,b), 30 μm (Figure 7c,d), 50 μm (Figure 7e,f), 70 μm (Figure 7g,h), 90 μm (Figure 7i,j), and flat LCE substrates (Figure 7k,l). On the 10 μm grooves, cells exhibited a strongly uniaxial morphology with nearly all cell bodies aligned parallel to the groove direction, as confirmed by the narrow, sharply peaked histogram centered at 0°. As groove width increased to 30 and 50 μm, cells remained predominantly aligned but displayed progressively broader angular distributions, indicating a gradual relaxation of the topographic confinement. On the 70 and 90 μm grooves, the histograms broadened further, with a noticeable population of cells oriented at oblique angles, reflecting the diminished geometric constraint imposed by wider channels. On the flat LCE film, cells spread isotropically with no preferred orientation, yielding a nearly uniform angular distribution across the full ±90° range.

These qualitative observations were quantitatively captured by the cell orientation angle distributions (Figure 7b,d,f,h,j,l) and the corresponding nematic order parameter *S* (Figure 8a), which quantifies the degree of alignment on a scale from 1 (perfect alignment) through 0 (random) to −1 (perpendicular alignment). The order parameter decreased monotonically with increasing groove width: *S* = 0.941 ± 0.006 (*W* = 10 μm), 0.826 ± 0.020 (*W* = 30 μm), 0.780 ± 0.033 (*W* = 50 μm), 0.751 ± 0.052 (*W* = 70 μm), and 0.551 ± 0.089 (*W* = 90 μm). The flat LCE control (*S* = −0.003 ± 0.032) yielded order parameters close to zero, confirming isotropic cell orientation in the absence of topographic cues on the flat LCE substrate. Dunnett’s post hoc comparisons confirmed that all grooved conditions exhibited significantly higher S values than the flat LCE control (*p* < 0.0001 for *W* = 10, 30, 50, 70, and 90 μm). The transition from strong alignment (*S* ≈ 0.75) to moderate alignment (*S* ≈ 0.55) occurred between *W* = 70 and 90 μm, a range that corresponds approximately to the characteristic spread length of individual H9c2 cells on adhesive substrates.

In addition to orientation, the groove topography significantly influenced cell elongation, as quantified by the aspect ratio (AR, Figure 8b). Because AR is a single-cell morphometric, the data are presented as violin plots to display the full distribution of individual cell measurements; the replicate means (±SD) reported below were used for statistical comparison. AR decreased systematically from 4.14 ± 0.20 on the 10 μm grooves to 3.09 ± 0.12 (*W* = 30 μm), 2.91 ± 0.18 (*W* = 50 μm), 2.83 ± 0.14 (*W* = 70 μm), and 2.61 ± 0.18 (*W* = 90 μm). The flat LCE control yielded AR = 2.02 ± 0.04. Dunnett’s post hoc comparisons confirmed that all grooved conditions exhibited significantly higher AR than the flat LCE control (*p* < 0.0001 for *W* = 10, 30, and 50 μm; *p* < 0.001 for *W* = 70 μm; *p* < 0.01 for *W* = 90 μm), indicating that even the widest grooves retained a measurable elongation effect relative to the unpatterned surface. This progressive trend reflects the relief of lateral geometric confinement with increasing groove width: when the groove width is comparable to or smaller than the characteristic cell dimension, the groove walls physically restrict cell spreading in the transverse direction, promoting uniaxial elongation along the groove axis and directing the assembly of actin stress fibers into parallel arrays. As the groove width increases and eventually exceeds the cell dimension, this geometric constraint is relieved, allowing multidirectional spreading and yielding lower aspect ratios. Although the AR decreased with increasing groove width and reached 2.61 ± 0.18 at W = 90 μm, it was still significantly higher than that of the flat LCE control (2.02 ± 0.04, *p* < 0.01), indicating that even the widest grooves retained a measurable elongation effect relative to the unpatterned LCE surface.

The cell spreading area exhibited an inverse correlation with the degree of alignment, increasing monotonically across the grooved substrates (Figure 8c): 358.7 ± 22.1 μm^2^ (*W* = 10 μm), 384.0 ± 10.5 μm^2^ (*W* = 30 μm), 427.8 ± 10.4 μm^2^ (*W* = 50 μm), 461.2 ± 35.5 μm^2^ (*W* = 70 μm), 509.9 ± 15.5 μm^2^ (*W* = 90 μm), and 609.2 ± 12.2 μm^2^ on the flat LCE control. Compared with the flat LCE control, the projected cell areas were significantly lower for *W* = 10, 30, 50, and 70 μm (*p* < 0.0001), and remained significantly lower for *W* = 90 μm (*p* < 0.001). On the LCE substrates, the confined groove geometry channels cytoskeletal polymerization predominantly along the groove axis on narrow substrates, reducing the total projected contact area; on wider grooves and the flat surface, cells extend lamellipodia more freely, yielding progressively larger but less anisotropic footprints. The spreading areas of the 10 and 30 μm groups were closely comparable, as were the 50 and 70 μm groups, suggesting that the area response to topographic confinement follows a stepwise rather than continuously graded pattern, with transitions occurring between *W* = 30–50 μm and *W* = 90 μm-Flat.

Cell density was additionally quantified and summarized in Table S3. Compared with the flat LCE control, cell density was significantly lower on the 10, 30, and 50 μm grooves (*p* < 0.01) and on the 70 μm grooves (*p* < 0.05), whereas no significant difference was observed for the 90 μm grooves. This trend indicates that narrow grooves may modestly limit cell attachment or packing, while the widest grooves support cell densities comparable to the flat LCE surface.

The comparison between LCE-based substrates and glass further provides a general reference for evaluating substrate cytocompatibility (Figure S15, Table S3). Although differences in spreading area and cell density were observed between the LCE substrates and glass, cells on both grooved and flat UCNPs/Azo-LCE substrates exhibited clear F-actin organization and intact nuclear morphology after 24 h of culture, while maintaining short-term cell viability above 85% over the 24 h culture period. These observations suggest that the UCNPs/Azo-LCE substrates can support H9c2 cell adhesion and spreading under the present culture conditions.

The coordinated trends observed across all four morphometric parameters-orientation angle, order parameter, cell area, and elongation ratio-are mutually consistent: narrow grooves simultaneously promote high alignment, strong elongation, and reduced projected footprint, while wide grooves and flat surfaces yield progressively isotropic and well-spread morphologies. Notably, the order parameter on the 10 μm grooves (*S* = 0.941) indicates near-perfect uniaxial alignment achieved through purely topographic cues, underscoring the potential of UCNPs/Azo-LCE substrates for directing cardiomyoblast organization.

Collectively, these results establish that microgroove-patterned UCNPs/Azo-LCE substrates can modulate the alignment, elongation, spreading area, and packing density of H9c2 cells in a groove width-dependent manner while showing no obvious signs of acute cytotoxicity over the 24 h culture period. The combination of tunable topographic guidance with NIR-responsive photomechanical actuation positions these substrates as a potential multifunctional platform for cardiac tissue engineering applications, where anisotropic cell alignment is a prerequisite for recapitulating the native myocardial architecture and achieving coordinated electromechanical coupling.

## 4 Conclusion

In summary, we developed an 808 nm-responsive Azo-LCE actuator by incorporating Nd^3+^-sensitized CSS-UCNPs into the LCE matrix to convert NIR light into local UV/blue emission for azobenzene isomerization. This design enabled repeatable photomechanical bending under 808 nm irradiation, with a maximum bending angle of 42.8 ± 2.6° at 24 W cm^–2^ and stable actuation over 50 cycles at 16 W cm^–2^. Notably, under biologically relevant irradiation intensities of 4–8 W cm^–2^, the system showed only a modest increase in the surrounding-medium temperature (Δ*T* = 2.29–4.36 °C) and no cumulative heating during repeated cycling. Moreover, microgroove-patterned UCNP/Azo-LCE substrates promoted H9c2 cell adhesion and spreading and guided groove-width-dependent uniaxial alignment. Together, these results establish a material platform that integrates NIR-responsive actuation with topographic guidance, offering a basis for future investigations of dynamic cell mechanoregulation.

## Supplementary Material





## Notes

Experimental details, synthesis and characterization of UCNPs, photomechanical and photothermal measurements, cell culture procedures, supplementary XRD, TEM, UV–vis, DSC, POM, UCL, infrared thermal, SEM, live/dead staining and fluorescence microscopy data, and supplementary tables of particle size, surface temperature, and cell morphology/alignment parameters (PDF)
Movie showing the reversible bending behavior of the UCNPs/Azo-LCE film under 808 nm NIR irradiation (MP4)
