# Supplementary material for: Near-Infrared Light-Driven Microgrooved UCNPs/Azobenzene-LCE Actuators and Substrates for Cardiomyoblast Alignment
Source: ACS Appl Mater Interfaces. 2026 Jul 4;18(28):39313–26. doi: 10.1021/acsami.6c07815 (PMC13397487; doi:10.1021/acsami.6c07815)
Supplement: Supplementary file 1 [file am6c07815_si_002.pdf]

Supporting information

Near-Infrared Light-Driven Microgrooved UCNPs/Azobenzene-LCE Actuators and Substrates for Cardiomyoblast Alignment

Chun Li[1], Zhenjia Huang[1,], Tongqing Li[1,], Petar S. Uskoković[2], Mo Yang[3], Linxia Gu[4], Gary Chi-Pong Tsui[1,]*

[1] Advanced Manufacturing Technology Research Centre, Department of Industrial and Systems Engineering, The Hong Kong Polytechnic University, Hong Kong, China.

[2] Faculty of Technology and Metallurgy, University of Belgrade, Karnegijeva 4, Belgrade, 11120, Serbia.

[3] Department of Biomedical Engineering, The Hong Kong Polytechnic University, Hong Kong, China.

[4] Department of Biomedical Engineering and Science, Florida Institute of Technology, Melbourne, 32901, FL, United States.

*Corresponding Author: Email Address: mfgary@polyu.edu.hk (Prof. Gary Chi-Pong Tsui),

Experimental Section

1 Materials

Ammonium fluoride (NH_4F), oleic acid (OA), sodium hydroxide (NaOH), 1-octadecene (ODE), neodymium(III) acetate hydrate ($(CH_3CO_2)_3Nd \cdot xH_2O$), thulium(III) acetate hydrate ($(CH_3CO_2)_3Tm \cdot xH_2O$), gadolinium(III) acetate hydrate ($(CH_3CO_2)_3Gd \cdot xH_2O$), ytterbium(III) acetate hydrate($(CH_3CO_2)_3Yb \cdot xH_2O$), yttrium(III) acetate hydrate ($(CH_3CO_2)_3Y \cdot xH_2O$), Irgacure 369, Triton X-100, fetal bovine serum (FBS), and bovine serum albumin (BSA) were purchased from Sigma-Aldrich. 4-Methoxybenzoic acid 4-(6-acryloyloxyhexyloxy) phenyl ester (C6BP), 1,4-bis[4-(3-acryloyloxypropyloxy) benzoyloxy]-2-methylbenzene (RM257) and 4,4'-bis[6-(acryloyloxy)hexyloxy] azobenzene (Azo) were purchased from SYNTHON Chemicals GmbH & Co. KG, Germany. The polyimide (PI)-based aligner (DL-2590) was purchased from Shenzhen Dalton Electronic Material Co., Ltd., China. Rat cardiomyoblast cells (H9c2 cells) and Calcein/PI Cell Viability/Cytotoxicity Assay Kit were purchased from Beyotime Biotechnology, China. High-glucose Dulbecco's Modified Eagle Medium (DMEM, high glucose), trypsin, 1% penicillin/streptomycin (P/S), 0.25% trypsin-EDTA, phosphate buffered saline (PBS, 1×, pH=7.4), paraformaldehyde (PFA, 4%), ProLong™ Glass Antifade Mountant with NucBlue™ Stain, Alexa Fluor™ 488 phalloidin, and dimethyl sulfoxide (DMSO) were purchased from Thermo Fisher Scientific. All chemicals were of analytical grade and used as received without further purification.

2 Synthesis and Characterizations of Core-shell-shell UCNPs

$NaYF_4$:20%Yb/0.5%Tm@$NaYF_4$:10%Yb/20%Nd@$NaYF_4$ CSS-UCNPs were synthesized via a modified three-step high-temperature coprecipitation method based on the protocol reported by Wang et al[48].

2.1 Synthesis of Hexagonal (β)-NaYF₄:Yb/Tm Core Nanoparticles

The core nanoparticles of $NaYF_4$:20%Yb/0.5%Tm (C-UCNPs) were synthesized by a thermal decomposition method. In a typical procedure, 0.805 mmol of $(CH_3CO_2)_3Y\cdot xH_2O$, 0.18 mmol of $(CH_3CO_2)_3Yb\cdot xH_2O$, and 0.005 mmol of $(CH_3CO_2)_3Tm\cdot xH_2O$ were added into a 100 mL three-necked flask containing 6 mL OA and 15 mL ODE. The mixture was heated to 120 ˚C under vigorous stirring to remove residual water and subsequently maintained at 150 ˚C for 1 h to form a transparent solution. After cooling down to room temperature, 10 mL methanol solution containing 2.5 mmol NaOH and 4 mmol NH_4F was added, and the mixture was maintained at 50 ˚C for 1 h. The methanol and residual water were then removed by sequentially heating to 80 ˚C and 120 ˚C, respectively. The mixture was subsequently heated to 300 ˚C at a rate of 10 ˚C min^{-1} and maintained for 1.5 h under a nitrogen atmosphere. After cooling to room temperature, the resulting $NaYF_4$:Yb/Tm nanoparticles were precipitated by the addition of ethanol, collected by centrifugation, washed with a mixture of ethanol and cyclohexane, and finally redispersed in 5 mL of cyclohexane.

2.2 Synthesis of NaYF₄:Yb/Tm@NaYF₄:Yb/Nd Core-Shell Nanoparticles

$NaYF_4$:20%Yb/0.5%Tm@$NaYF_4$:10%Yb/20%Nd core-shell nanoparticles (CS-UCNPs) were synthesized by an epitaxial shell growth approach. Specifically, 0.7 mmol of $(CH_3CO_2)_3Y\cdot xH_2O$, 0.1 mmol of $(CH_3CO_2)_3Yb\cdot xH_2O$, and 0.2 mmol of $(CH_3CO_2)_3Nd\cdot xH_2O$ were added into a 100

mL three-necked flask containing 6 mL OA and 15 mL ODE. After removing residual water at 120 ˚C, the mixture was maintained at 150 ˚C for 1 h to form a transparent solution. After cooling to room temperature, 10 mL of methanol solution containing 4 mmol NH_4F and 2.5 mmol NaOH, along with the as-prepared $NaYF_4$:Yb/Tm core nanoparticles dispersed in cyclohexane, were added and maintained at 50 ˚C for 30 min. The methanol, cyclohexane, and residual water were subsequently removed by heating to 80 ˚C and 120 ˚C, respectively. The mixture was then heated to 300 ˚C at a rate of 10 ˚C min^{-1} and maintained for 1.5 h. After cooling to room temperature, the resulting CS-UCNPs were precipitated by the addition of ethanol, collected by centrifugation, washed with a mixture of ethanol and cyclohexane, and redispersed in 5 mL of cyclohexane.

2.3 Synthesis of $NaYF_4$:Yb/Tm@$NaYF_4$:Yb/Nd@$NaYF_4$ Core-Shell-Shell Upconversion Nanoparticles

To synthesize the $NaYF_4$:20%Yb/0.5%Tm@$NaYF_4$:10%Yb/20%Nd@$NaYF_4$ CSS-UCNPs, 1 mmol $(CH_3CO_2)_3Y{\cdot}xH_2O$ was added into a 100 mL three-necked flask containing 6 mL OA and 15 mL ODE. After removing residual water at 120 ˚C, the mixture was maintained at 150 ˚C for 1 h to form a transparent solution. After cooling to room temperature, 10 mL of methanol solution containing 4 mmol NH_4F and 2.5 mmol NaOH, along with the as-prepared CS-UCNPs dispersed in cyclohexane, were added and maintained at 50 ˚C for 30 min. The methanol, cyclohexane, and residual water were subsequently removed by heating to 80 ˚C and 120 ˚C, respectively. The mixture was then heated to 300 ˚C at a rate of 10 ˚C min^{-1} and maintained for 1.5 h. After cooling to room temperature, the resulting CSS-UCNPs were precipitated by the addition of ethanol,

collected by centrifugation, washed with a mixture of ethanol and cyclohexane, and redispersed in 5 mL of cyclohexane for further use.

3 Characterizations of the UCNPs and the UCNPs/Azo-LCE Films

The morphology of the synthesized UCNPs was examined by transmission electron microscopy (TEM, JEOL JEM-2010) at an acceleration voltage of 120 kV. The particle size distribution of the CSS-UCNPs was determined by measuring at least 100 individual nanoparticles from TEM micrographs using ImageJ software. The upconversion luminescence (UCL) spectra of the UCNPs were recorded on a fluorescence spectrophotometer (Edinburgh FLS980) in the wavelength range of 200–800 nm under 808 nm CW NIR laser irradiation at a power of 600 mW. The crystalline phase of the UCNPs was characterized by X-ray diffraction (XRD, Rigaku SmartLab 9kW-Advance) using Cu Kα radiation at room temperature over a 2θ range of 10–80° with a scanning rate of 2° min^{-1}. The UV-Vis-NIR absorption spectra of the Azo and UCNP/Azo solutions were acquired on a UV-Vis-NIR spectrometer (PerkinElmer, Lambda 1050) in the wavelength range of 200–800 nm. The thermal properties of the UCNPs/Azo-LC precursor and the cured LCE films were investigated by differential scanning calorimetry (DSC, Mettler Toledo DSC3) under a nitrogen flow in the temperature range of 0–120 °C at a heating rate of 10 °C min^{-1}.

For photomechanical actuation measurements, the LCE film strips were irradiated with an 808 nm CW NIR laser (0–10 W) at normal incidence to induce bending deformation. The laser has a fixed rectangular spot of 4.8 × 5.2 mm^2. The laser was positioned at a distance of 15 cm from the sample surface. Recovery occurred spontaneously upon removal of the NIR irradiation. The

bending angle (θ) was defined as the angle between the horizontal baseline and the line connecting the midpoint of the fixed end and the midpoint of the free end of the bent film; the maximum value attained during deformation was denoted as θ_{max}. For each measurement, the bending angles on both sides of the film were measured and averaged as θ. For each power density, four independent samples were tested ($n = 4$), and the results are reported as mean ± standard deviation (SD). The deformation process was recorded using a digital camera.

4 Cell culture

All cells were cultured in complete medium consisting of 89% high-glucose DMEM, 10% FBS, and 1% penicillin-streptomycin, and maintained at 37 °C in a humidified incubator with 5% CO_2. Prior to cell seeding, the LCE film samples were sterilized by immersion in 75% ethanol for 1 h, followed by three washes with PBS for 15 min each. H9c2 cells were detached using 0.25% trypsin-EDTA, resuspended in complete medium, and seeded directly onto the LCE films at a density of approximately 1.63×10^4 cells cm^{-2}.

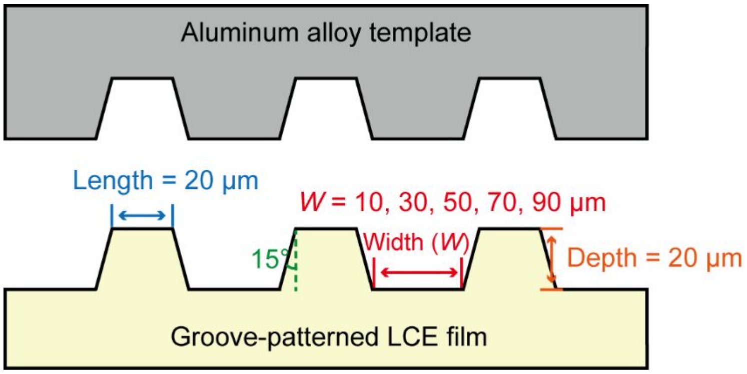

Figure S1. Schematic cross-sectional illustration of the microgroove fabrication process. An aluminum alloy template (top, gray) with periodic groove features serves as the mold. After LC mixture infiltration, alignment, and UV photopolymerization, the cured LCE film (bottom, yellow) is peeled off, producing a groove-patterned surface. Key geometric parameters: ridge width = 20 μm, groove depth = 20 μm, groove width (*W*) = 10, 30, 50, 70, and 90 μm. The groove sidewalls exhibit a draft angle of ~15˚.

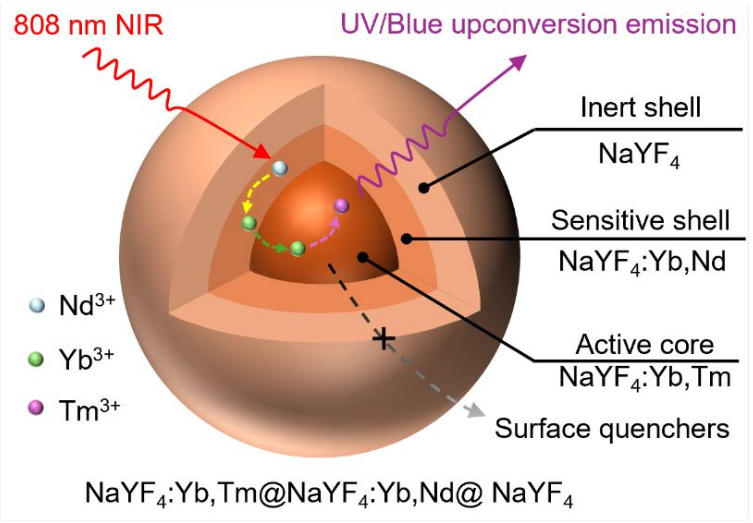

Figure S2. Schematic diagram of the CSS-UCNPs structure

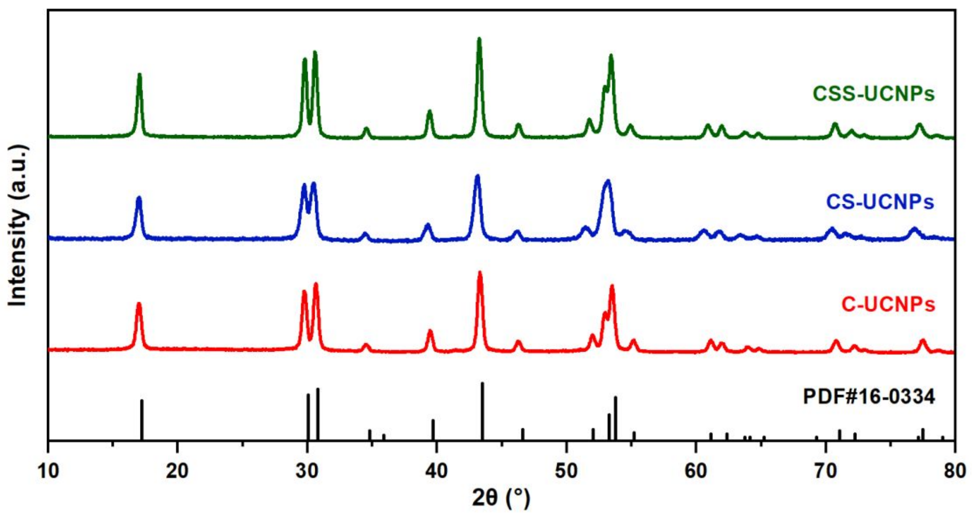

Figure S3. XRD spectra of C-UCNPs, CS-UCNPs, and CSS-UCNPs with reference to the standard PDF#16-0334.

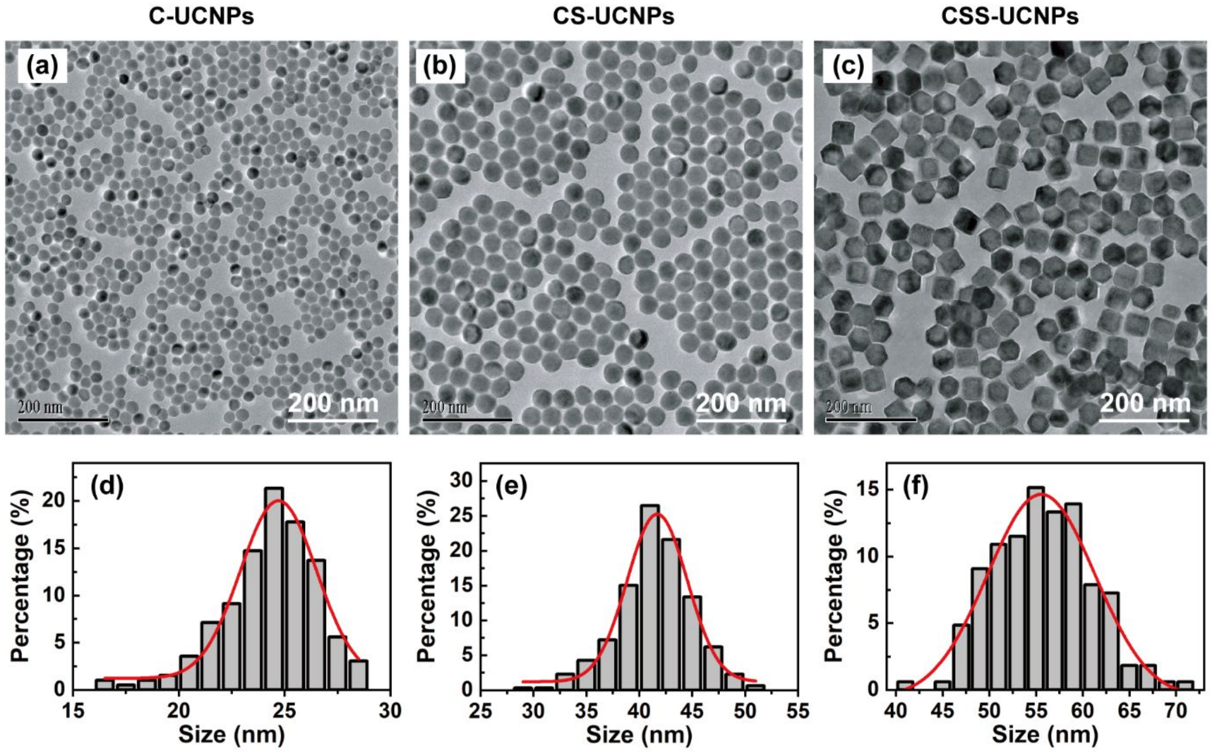

Figure S4. TEM images and particle size distribution diagrams of C-UCNPs, CS-UCNPs, and CSS-UCNPs.

Table S1. Average particle sizes of C-UCNPs, CS-UCNPs, and CSS-UCNPs

	Size (nm)
C-UCNPs	24.3 ± 2.2
CS-UCNPs	41.5 ± 3.5
CSS-UCNPs	55.7 ± 5.3

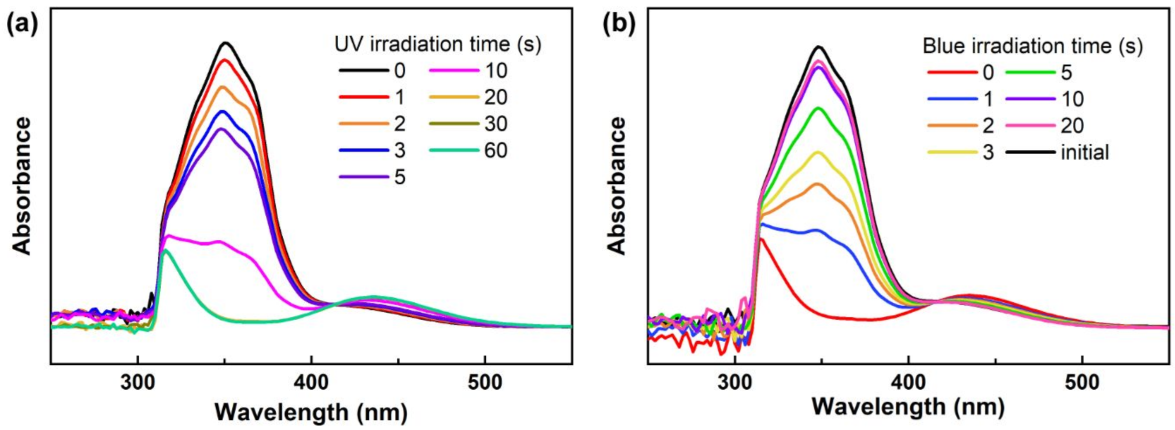

Figure S5. (a) UV-Vis spectra of *trans*-Azo after exposure to UV light (365 nm, 20 W) for different durations; (b) UV-Vis spectra of *cis*-Azo after exposure to blue light (450 nm, 10 W) for different durations. The concentration of Azo is 0.5 mg mL^{-1} in acetone.

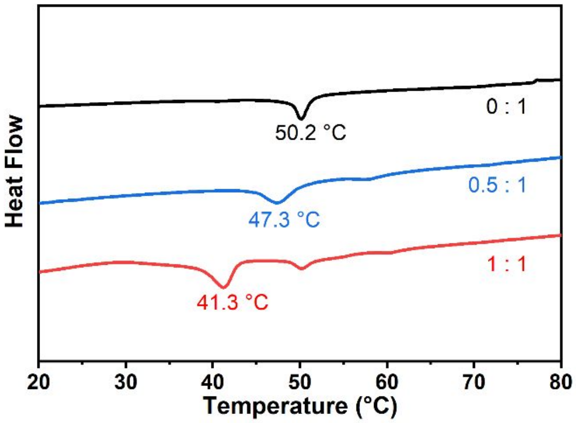

Figure S6. DSC thermograms of LC mixtures with different UCNP-to-Azo weight ratios.

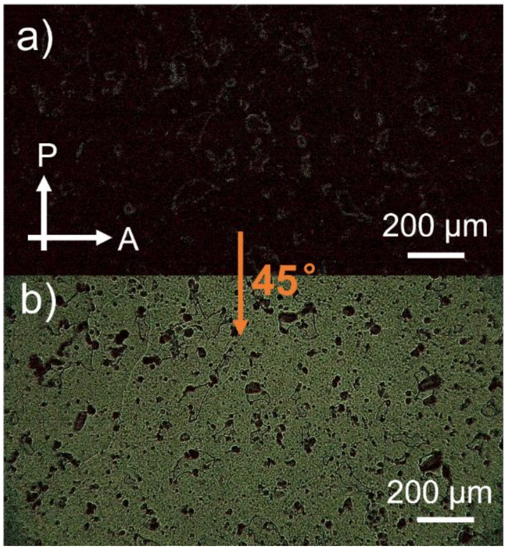

Figure S7. Polarized optical microscopy (POM) images of the UCNPs/Azo-LCE film under crossed polarizers. (a) The film oriented with the liquid crystal director parallel to the polarizer (P), showing near-complete extinction, indicative of uniform molecular alignment. (b) Upon rotation of the sample by 45° relative to the crossed polarizers, strong birefringence is observed, confirming the homogeneous nematic order within the LCE network. Scale bars: 200 µm.

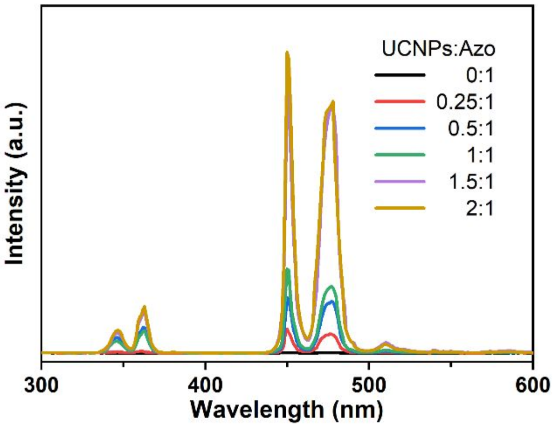

Figure S8. UCL spectra of UCNPs/Azo-LCE films with different UCNPs:Azo ratios under 808 nm excitation.

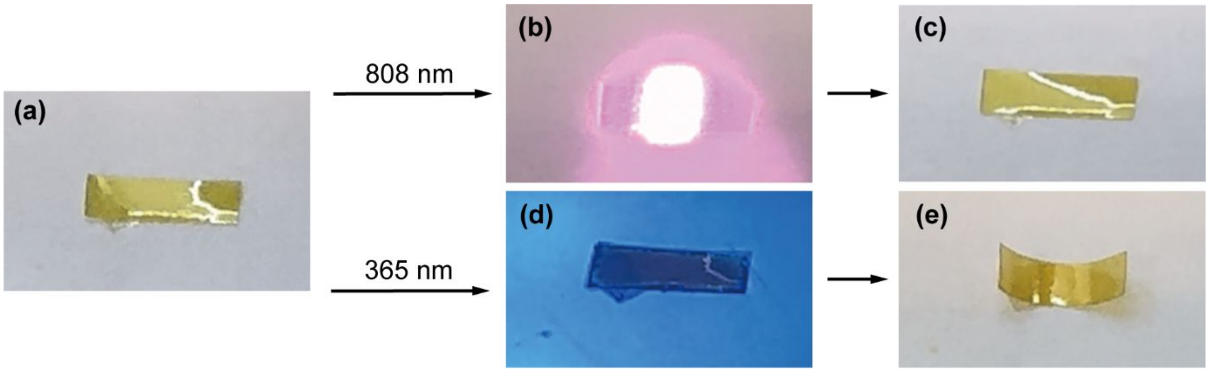

Figure S9. Bending response of the Azo-LCE film (without UCNPs) under 808 nm NIR (16 W cm^{-2}) and 365 nm UV irradiation (20W). (a) Digital photograph of the film in the initial flat state. (b, c) Film under 808 nm NIR laser irradiation (b) and after irradiation (c), showing no observable bending deformation, confirming that without UCNPs, NIR light alone cannot trigger the *trans*-to-*cis* isomerization of azobenzene crosslinkers. (d, e) Film under 365 nm UV irradiation (d) and after irradiation (e), exhibiting significant bilateral bending deformation, demonstrating that direct UV light can effectively induce azobenzene photoisomerization and drive macroscopic actuation of the Azo-LCE film.

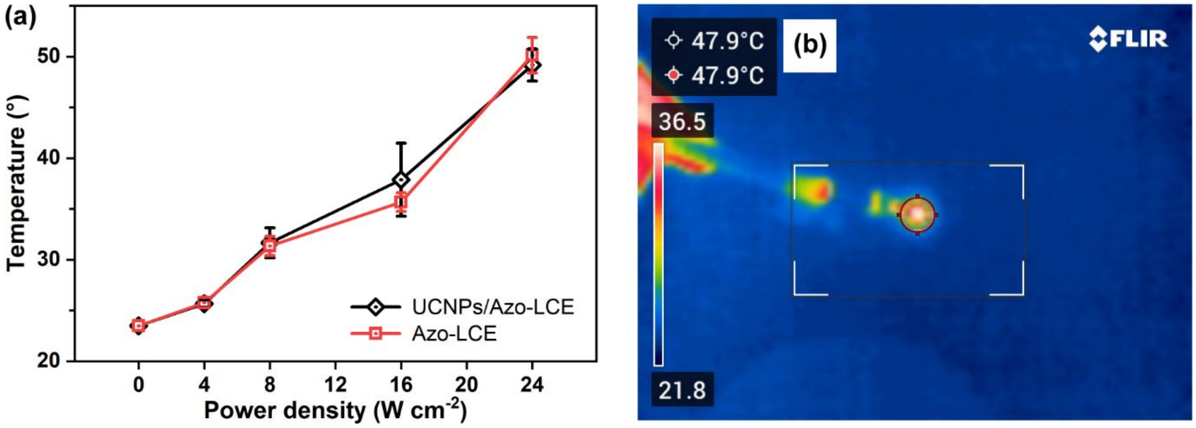

Figure S10. (a) Surface temperature of the UCNPs/Azo-LCE composite film and the Azo-LCE film as a function of 808 nm NIR laser power density. (b) Representative thermal infrared images of the UCNPs/Azo-LCE composite film recorded during 808 nm CW laser irradiation at a power density of 24 W cm^{-2}.

Table S2. Surface temperature of UCNPs/Azo-LCE and Azo-LCE films under 808 nm NIR irradiation.

Power density ($W\ cm^{-2}$)	Temperature (˚C)	
	UCNPs/Azo-LCE	Azo-LCE
4	25.68 ± 0.36	25.78 ± 0.51
8	31.67 ± 1.49	31.36 ± 0.95
16	37.88 ± 3.59	35.66 ± 0.91
24	49.16 ± 1.57	50.14 ± 1.75

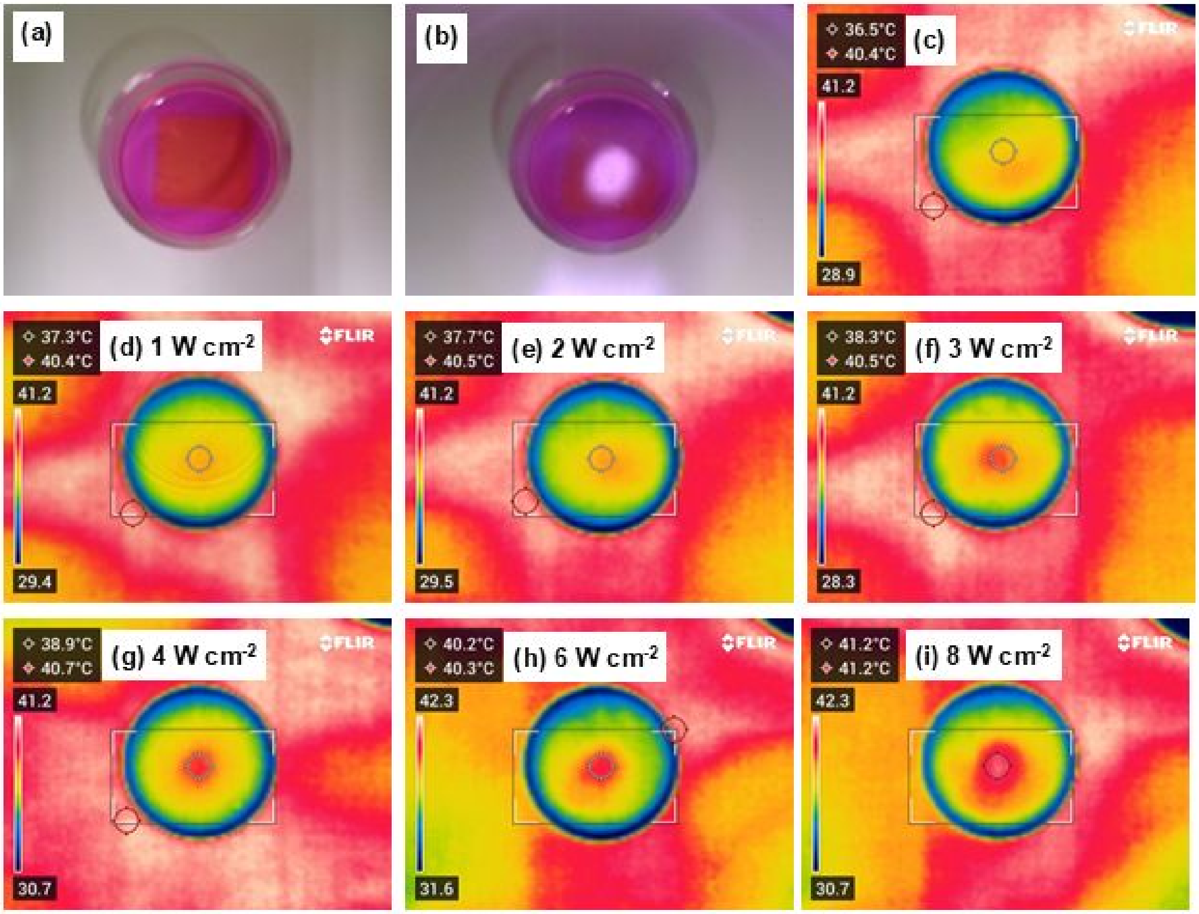

Figure S11. Representative infrared thermal images during photothermal characterization under cell-culture-mimicking conditions. (a, b) Digital photographs of the film with NIR laser off (a) and on (b); the oblique incidence of the circular beam produces an elliptical spot (~0.9 × 1.2 cm^2) on the film surface. (c) Infrared thermal image of the film at ambient conditions. (d–i) Steady-state thermal images captured after 10 min of continuous 808 nm irradiation at power densities of 1 (d), 2 (e), 3 (f), 4 (g), 6 (h), and 8 W cm^{-2} (i). All thermal images were recorded using a FLIR infrared camera.

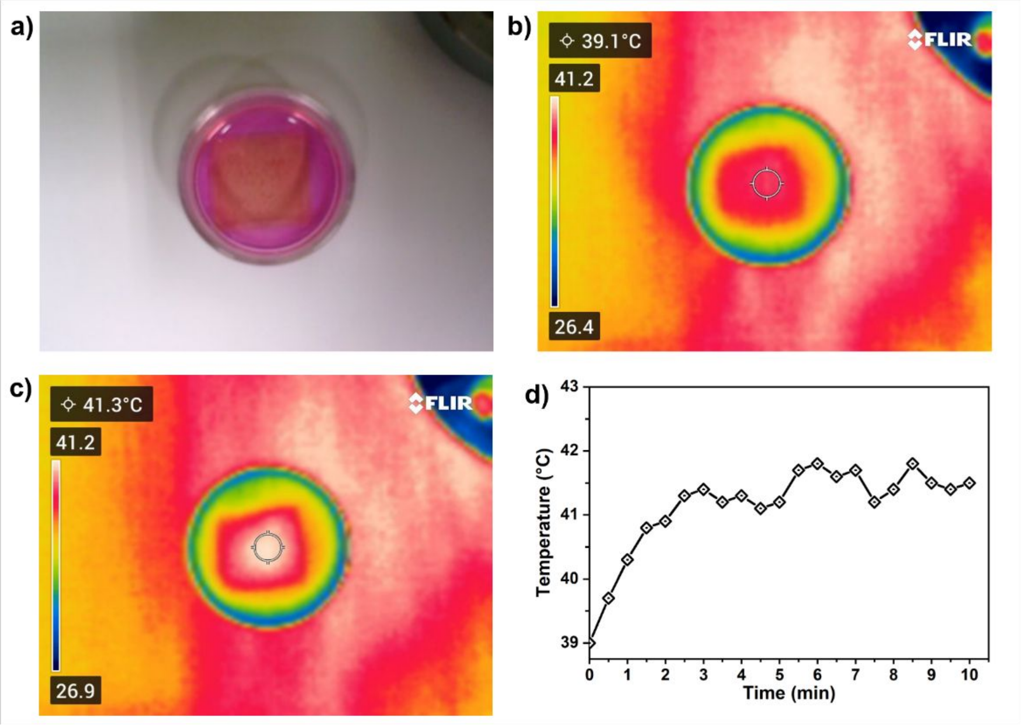

Figure S12. Supplementary thermal assessment of the UCNPs/Azo-LCE film surface using a floating-film configuration. (a) Schematic illustration of the experimental setup, in which the UCNPs/Azo-LCE film was floated on the surface of 2 mL of culture medium in a 35 mm glass-bottom dish maintained at 37 ˚C, and the upper film surface was monitored by infrared thermal imaging during 808 nm irradiation. (b) Representative infrared thermal image before irradiation. (c) Representative infrared thermal image during irradiation at 8 W cm^{-2} for 10 min. (d) Surface temperature of the floating UCNPs/Azo-LCE film as a function of irradiation time under 8 W cm^{-2}.

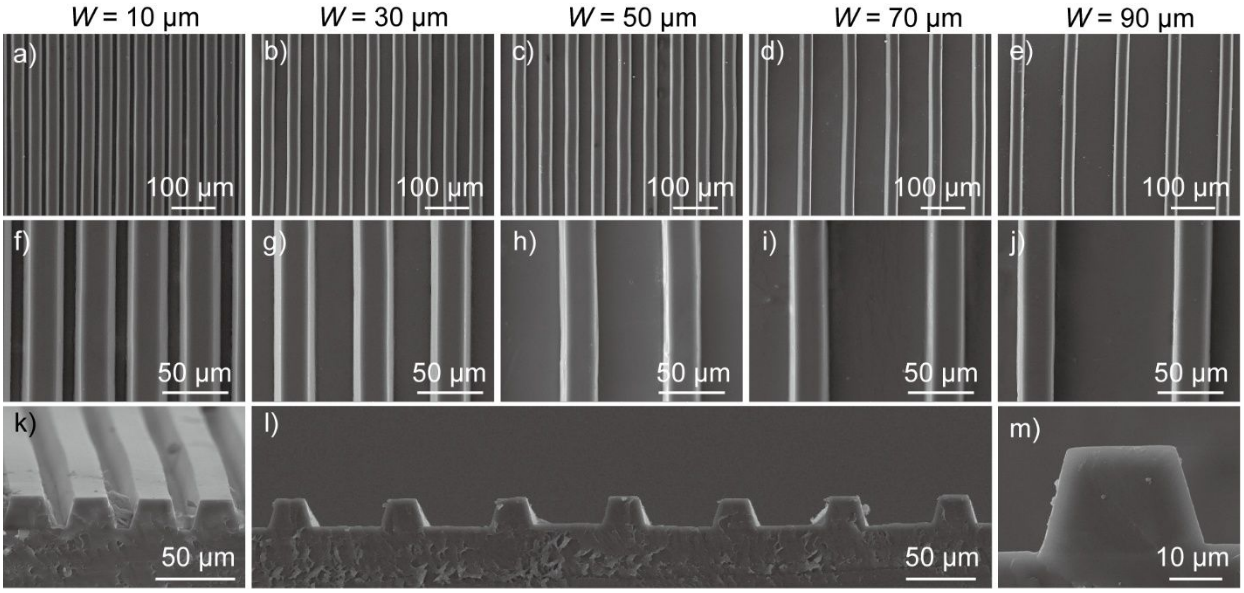

Figure S13. SEM characterization of microgroove-patterned UCNPs/Azo-LCE films. From left to right, the groove widths are W = 10, 30, 50, 70, and 90 μm. (a–e) Top-view SEM images acquired at 500× magnification. (f–j) Corresponding top-view SEM images acquired at 1500× magnification. Scale bars: 100 μm in (a–e) and 50 μm in (f–j). (k) Cross-sectional SEM image of the groove structure with W = 10 μm, showing an inclined sidewall. Scale bar: 50 μm. (l) Cross-sectional SEM image of the groove structure with W = 50 μm. Scale bar: 50 μm. (m) Magnified cross-sectional SEM image of a single groove. Scale bar: 10 μm.

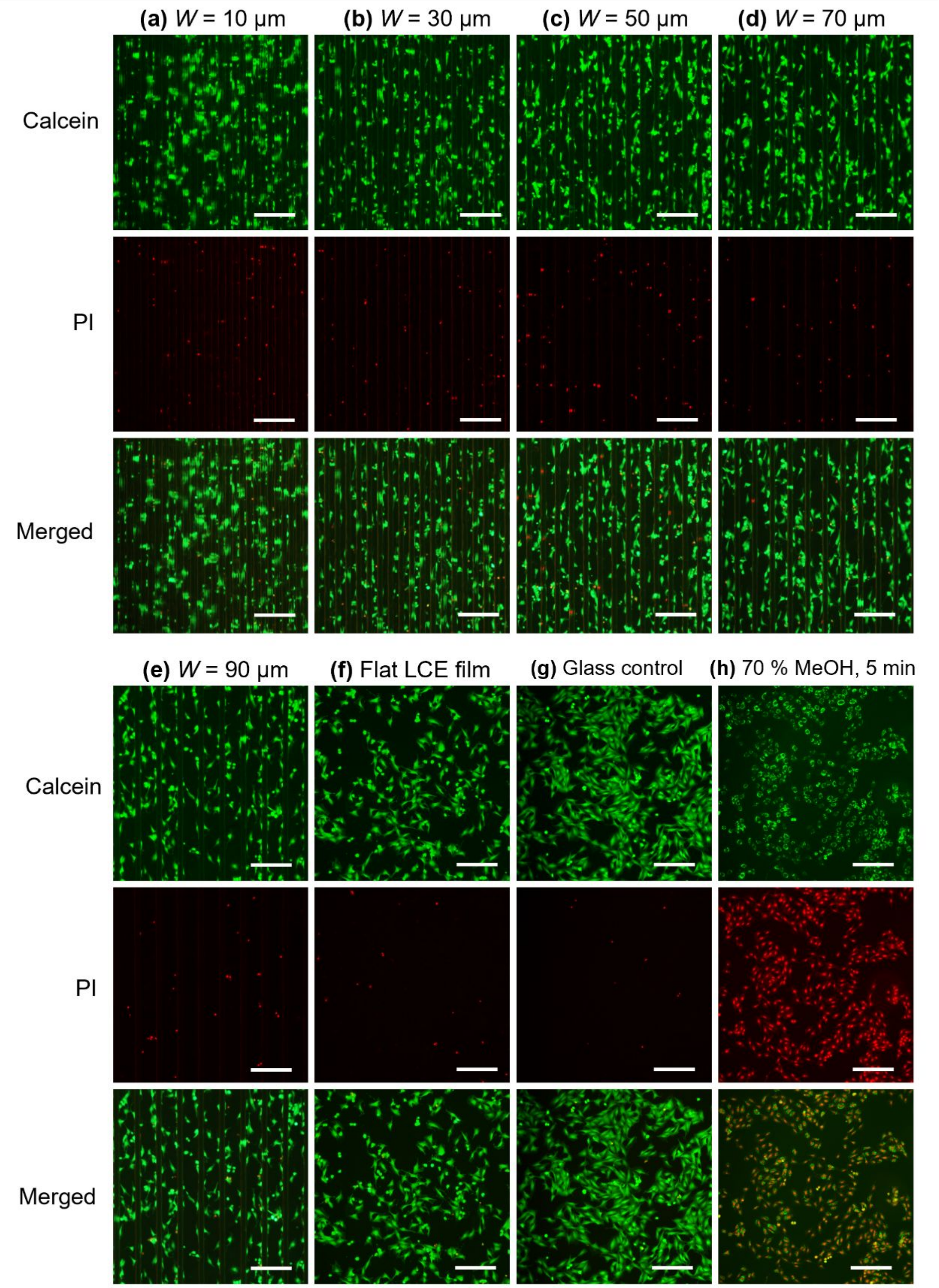

Figure S14. Representative Calcein AM/PI live/dead staining images of H9c2 cells cultured on different substrates for 24 h. For each substrate condition, three panels are shown: Calcein AM

(green, live cells), PI (red, dead cells), and merged (overlay). (a) W = 10 μm, (b) 30 μm, (c) 50 μm, (d) 70 μm, (e) 90 μm, (f) flat LCE film, (g) negative control (glass coverslip), (h) positive control (cells on glass treated with 70% methanol for 5 min). Scale bar: 200 μm (applies to all panels).

Table S3. Summary of Mean ± SD values for the nematic order parameter (*S*), cell aspect ratio (AR), projected cell area and cell density

Width (μm)	Nematic order parameter *S*	Cell aspect ratio (AR)	Projected cell spreading area (μm^2)	Cell Density (cell mm^{-2})
10	0.941 ± 0.006	4.14 ± 0.20	358.7 ± 22.1	348.5 ± 14.6
30	0.826 ± 0.020	3.09 ± 0.12	384.0 ± 10.5	342.3 ± 21.3
50	0.780 ± 0.033	2.91 ± 0.18	427.8 ± 10.4	349.6 ± 15.3
70	0.751 ± 0.052	2.83 ± 0.14	461.2 ± 35.5	365.2 ± 23.7
90	0.551 ± 0.089	2.61 ± 0.18	509.9 ± 15.5	390.8 ± 37.2
∞	-0.003 ± 0.032	2.02 ± 0.04	609.2 ± 12.2	428.2 ± 30.7
glass	-0.052 ± 0.059	1.83 ± 0.04	852.6 ± 30.1	850.1 ± 50.2

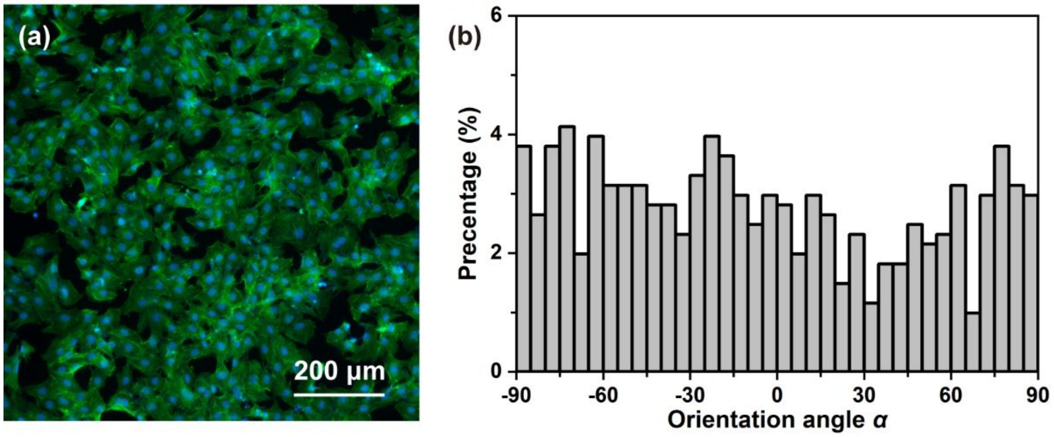

Figure S15. (a) Fluorescence microscopy images on glass substrates. F-actin is stained with Alexa Fluor 488 phalloidin (green) and nuclei with DAPI (blue). Scale bars: 200 μm. (b) The angular distribution histogram shows the orientation angle α of cells relative to the groove direction. The x-axis represents the orientation angle α (°), and the y-axis represents the percentage of cells in each angular bin, calculated as the number of cells in a given bin divided by the total number of cells.
